# Multilevel model for airborne transmission of foot-and-mouth disease applied to Swedish livestock

**DOI:** 10.1371/journal.pone.0232489

**Published:** 2020-05-26

**Authors:** Oscar Björnham, Robert Sigg, Jan Burman

**Affiliations:** Swedish Defence Research Agency, Umeå, Sweden; Arizona State University & Santa Fe Institute, UNITED STATES

## Abstract

The foot-and-mouth disease is an ever-present hazard to the livestock industry due to the huge economic consequences following an outbreak that necessitates culling of possibly infected animals in vast numbers. The disease is highly contagious and previous epizootics have shown that it spreads by many routes. One such route is airborne transmission, which has been investigated in this study by means of a detailed multilevel model that includes all scales of an outbreak. Local spread within an infected farm is described by a stochastic compartment model while the spread between farms is quantified by atmospheric dispersion simulations using a network representation of the set of farms. The model was applied to the Swedish livestock industry and the risk for an epizootic outbreak in Sweden was estimated using the basic reproduction number of each individual livestock-holding farm as the endpoint metric. The study was based on comprehensive official data sets for both the current livestock holdings and regional meteorological conditions. Three species of farm animals are susceptible to the disease and are present in large numbers: cattle, pigs and sheep. These species are all included in this study using their individual responses and consequences to the disease. It was concluded that some parts of southern Sweden are indeed preconditioned to harbor an airborne epizootic, while the sparse farm population of the north renders such events unlikely to occur there. The distribution of the basic reproduction number spans over several orders of magnitudes with low risk of disease spread from the majority of the farms while some farms may act as very strong disease transmitters. The results may serve as basic data in the planning of the national preparedness for this type of events.

## Introduction

Cloven-hoofed animals are susceptible to the foot-and-mouth disease (FMD) which is highly contagious. The disease is rarely lethal but constitutes a severe threat to the livestock sector due to the economic impact that follows an outbreak. FMD is widely spread and is endemic in large parts of Africa and Asia [1]. It has also been endemic in South America since the 19th century, but as a result of comprehensive and successful efforts the disease is now close to being eradicated [2–4]. Regions considered free from FMD are occasionally exposed to the disease due to import of infected animals or contaminated products. Since the FMD virus (FMDV) is extremely infectious, the introduction of the virus in otherwise free regions may give rise to a rapid spread of the disease amongst susceptible animals, which is called an epizootic. Large-scale outbreaks of FMD have occurred a multitude of times on many continents the last century. A few significant cases may be highlighted. The 1924 Michigan outbreak lasted for two years and lead to the slaughter of over 170 000 animals [5]. UK was hit by an outbreak in 1967 resulting in the slaughter of almost 500 000 animals [6]. In 1981 an airborne epizootic outbreak took place in France and UK [7, 8] spreading over the British Channel. Taiwan was hit by a devastating wave of FMD spread within the pig population 1997, almost 4 million pigs were slaughtered to combat the epizootic [9]. Even though UK had already experienced heavy losses due to FMD at previous outbreaks, the kingdom was afflicted once again in 2001. This devastating foot-and-mouth epizootic resulted in the slaughter of over 6 million animals and the event was estimated to have cost over 9 billion US dollars [10, 11]. The 2001 UK epizootic triggered a vast amount of research concerning various aspects of FMD in general and investigations of the specific event in particular.

It is well established that FMD is capable of spreading amongst livestock by several different routes [12]. The, by far, most investigated type is the manual transport of animals or contaminated animal products which also seems to be the most prominent disease spread route in today´s societies [13–17]. Animals are transported for different reasons between farms or to common grounds, which may cause a rapid spread of the disease over great distances. A standard procedure to hinder an initiated epizootic is to proclaim an animal transportation ban. This is an effective and necessary action. However, as mentioned, the disease may spread through other routes. One of these routes is the airborne transport of aerosols containing the virus. Investigations into historical epizootics show that airborne transmission is undeniably responsible for part of the disease spread in many epizootics and numerical models have shown agreement with that conclusion. For instance, the 1967 UK outbreak [18], the 1981 UK/France outbreak [7, 8] and the UK 2001 outbreak [19–22] all included airborne transmission. Although transports of animals and animal products can be stopped by administrative measures that set an infected farm in quarantine, the atmospheric transmission is still present and constitutes a considerable risk for further disease spread [21]. Such events have frequently been observed and airborne transmission between farms constitutes an important route for FMD. It has most commonly been reported to occur over distances up to 10 km [19, 21, 23, 24] but there are also cases with considerably larger distances. For instance, Hugh-Jones et al. reported cases of transmission with distances exceeding 60 km during the 1967–1968 FMD outbreak in the UK [18]. It is difficult to dismiss other vectors such as wildlife, but the current meteorological conditions support that atmospheric transmission was, indeed, responsible. Disease spread by air will induce a wave-like spreading pattern over the geographical domain. One example is the spread of the H7N7 virus between poultry farms 2003 in the Netherlands [25].

In this study, we present a complete and highly detailed model design for the estimation of the probability of an airborne epizootic over a distributed set of farms. By utilizing data from several external studies that describe different properties of the disease, we collected all tools required to assemble a model of a possible epizootic driven by atmospheric transmission. The study is applied to Sweden and its livestock using the official statistics of the current stand of Swedish farms both with regard to the locations of the farms and the amount of animals of relevant species on each farm. A proficient atmospheric dispersion model was utilized to estimate the probability of transmission between farms. The airborne transmission takes the anisotropic reality (i.e. dependent on the direction) into consideration by incorporating meteorological conditions based on empirical historical data from the network of meteorological stations distributed over Sweden. The model incorporates a detailed description of the entire process on all scales and a Monte Carlo approach to quantify statistical and geographical distributions of the basic reproduction number, which serve as indicators for the risk for an epizootic. Even though Swedish livestock is addressed in this study, the basic methodology presented here is applicable to any other geographic area as well. The parameters used in this analysis are based on serotype O since it has occurred most frequently in European outbreaks and it has been clinically investigated in most detail.

The main features of the model is presented in the section “Methods”, while some more technical properties are collected in the appendix “Model properties”. The appendix includes a numerical study of the role of the number of iterations and time step length on the accuracy and precision, four case studies and a sensitivity analysis.

## Methods

Epizootics typically involve actions on different scales. The first scale addresses individual animals and the development of the disease for infected ones. The second scale handles all animals on a farm and models the disease spread amongst them. Finally, the third scale includes many farms in an arbitrary sized region and treats the epizootic spread over geographical distances.

**Fig 1:**
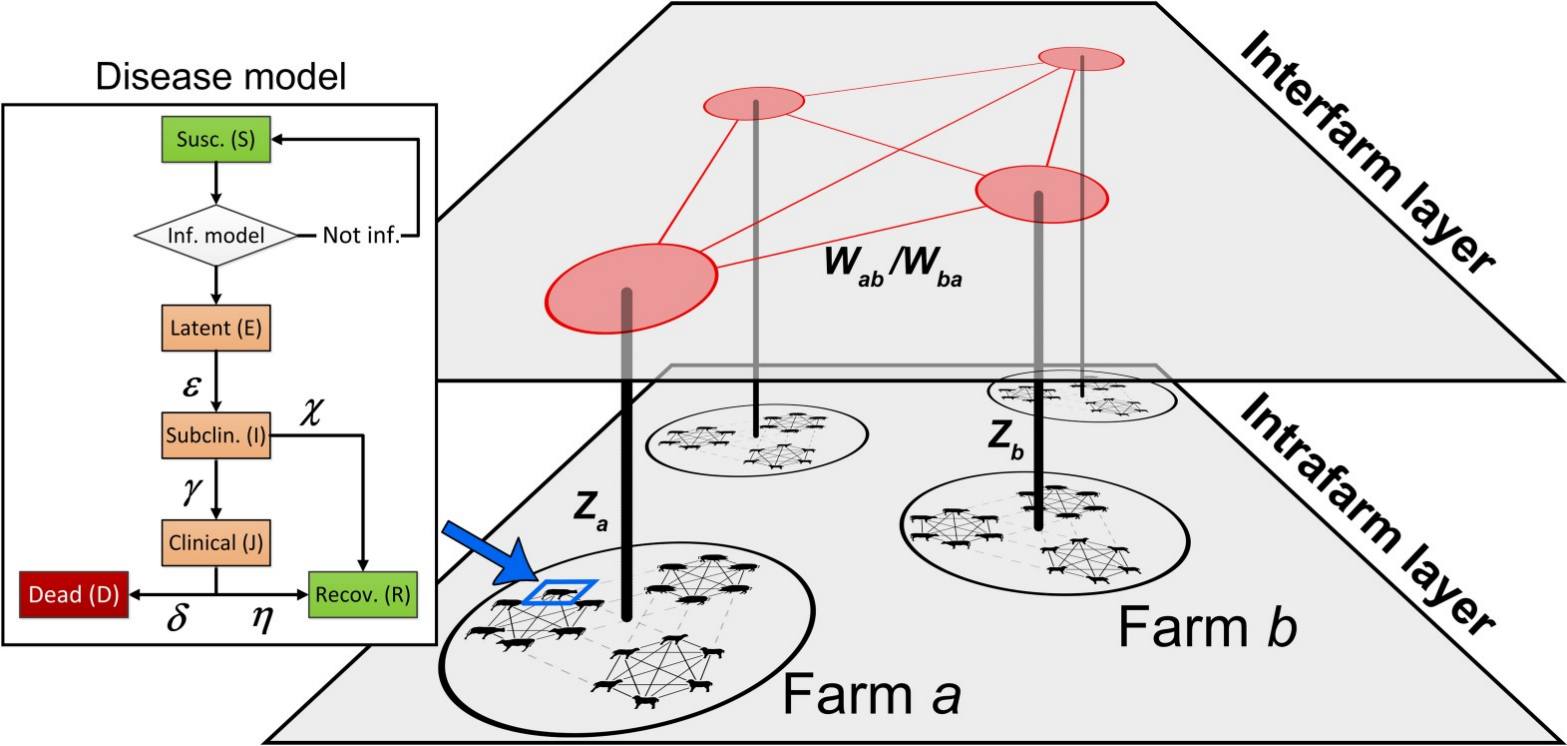
Schematic illustration of the multilevel model design. The epizootic model uses two layers. Individual animals are resolved on the intrafarm layer, where disease spread acts between animals on the same farm. The disease model for individual animals, visible on the left in the figure, is described in **Fig 2**. In contrast, the smallest resolved units on the interfarm layer are the farms. At this layer, the disease is spread between farms by atmospheric transmission.

In this study we have distributed the different disease spread components onto two different layers. The two layers operate under different modelling schemes, which allows all scales to be resolved adequately. Individual animals and local spread on farms take place on the *intrafarm layer* where there is no contact between the farms. This layer utilizes a compartment representation of the disease states for individual animals coupled to disease transmission between animals, which yields a closed stochastic equation system. Each infected farm has a source term, *Z*, that may cause an airborne disease spread between farms at the *interfarm layer* using the connection strengths *W*. The set of farms forms a weighted and directed network where the connection strength describes the probability of disease spread from one farm to another based on their geographic locations and the prevailing meteorological conditions in that area. Fig 1 illustrates the design for the epizootic model. Details for the three different scales are given separately below.

### Individual animals

#### Infection model

Infection models can be divided into two different basic types. The first type of models are deterministic which means that an infection will occur once, but not before, a certain threshold of pathogens is reached at the appropriate site within the animal. This does not strictly mean that several animals exposed to the same environment will all become infected simultaneous since there might be individual variability regarding the transfer probability to these appropriate sites. Deterministic models have been losing in popularity over time and they are often regarded as obsolete nowadays [26–29]. The second type of models are stochastic which means that any non-zero exposure may initiate an infection. The magnitude of the exposure will affect the probability of infection, i.e. there is no discrete threshold. The stochastic approach allows a single pathogen to cause infection and it is therefore often referred to as the *single-hit model*. Strong support for the stochastic model can be found in the literature [30–32].

Another factor that defines the infection model is whether the model assumes independent or synergetic pathogens. Independent pathogens will be modelled to act completely independent of each other, implying that the probability of one particular pathogen to cause infection does not depend on any other present pathogen. A more complicated nonlinear behavior with the dose is considered in a synergetic system. Little support have been found for any strong synergy in the infection [33].

Following the recommendations of the mainstream modelling in the literature, we use a stochastic model and assume independency between the pathogens. Applying the principles of the single-hit model, there is a probability that one pathogen causes an infection. Let *s* denote that probability, implying that 0 ≤ *s* ≤ 1. This parameter is referred to as the susceptibility of the animal for this type of pathogen. To avoid becoming infected by *n* pathogens, the animal must avoid becoming infected by each pathogen. Since the pathogens are assumed independent, the probability of infection from *n* pathogens becomes

$$P_{\inf}\left(n\right)=1-{\left(1-s\right)}^{n}.$$ (1)

If the pathogens can be considered to be independently spatially distributed then the inhaled number of pathogens is well described by the Poisson distribution, and the infection probability can be reformulated as Eq (2) which is referred to as the *exponential model* [34].

$$P_{\inf}\left(n\right)=1-e^{-ns}$$ (2)

#### Animal disease model

Mardones et al. performed a comprehensive review of the scientific literature regarding the duration of the different disease stages (corresponding to *states* here) associated with FMD [35]. They used experimental data from 19 different publications adding up to 295 animals in total. By defining the disease model to be consisting of four different stages (latent, subclinical, incubation, and infectious) they found the best fit of several investigated distributions for the duration of each stage and for three animal species: Cattle, Pigs and Small ruminants (Sheep). We note that the stages used by Mardones et al. are not consistently identical to the states used here. The terms *latent* and *subclinical* are indeed identical, while the definitions of *clinical* differ. However, the stage *infectious* as defined by Mardones et al. is identified to equal the sum of the *subclinical* state and *clinical* state as defined in this study, i.e. the state clinical ends when the animal is no longer infectious. Table 1 shows the reported mean duration times for the stages of interest and the calculated mean values for the clinic state.

**Table 1:** Disease model parameters for the duration of different disease stages (states).

Disease stage	Species	Mean	Disease stage	Species	Mean
**Latent**	Cattle	3.6	**Infectious**	Cattle	4.4
	Pigs	3.1		Pigs	5.7
	Sheep	4.8		Sheep	3.3
**Subclinical**	Cattle	2.0	**Clinical**	Cattle	2.4
	Pigs	2.3		Pigs	3.4
	Sheep	2.2		Sheep	1.1

The duration of the clinical state has been defined to equal the duration of the infectious stage minus the duration of the subclinical stage.

The mortality rate is reported to be <5% [35] for adult animals of all species while younger animals experiences a considerable higher mortality rate [36–38]. Animals that recover from infection profit from immunity for that specific serotype for a time period that exceeds the duration of a typical epizootic outbreak [39]. This means that these animals are effectively removed from the epizootic system when leaving the clinical state no matter if they die or recover.

**Fig 2:**
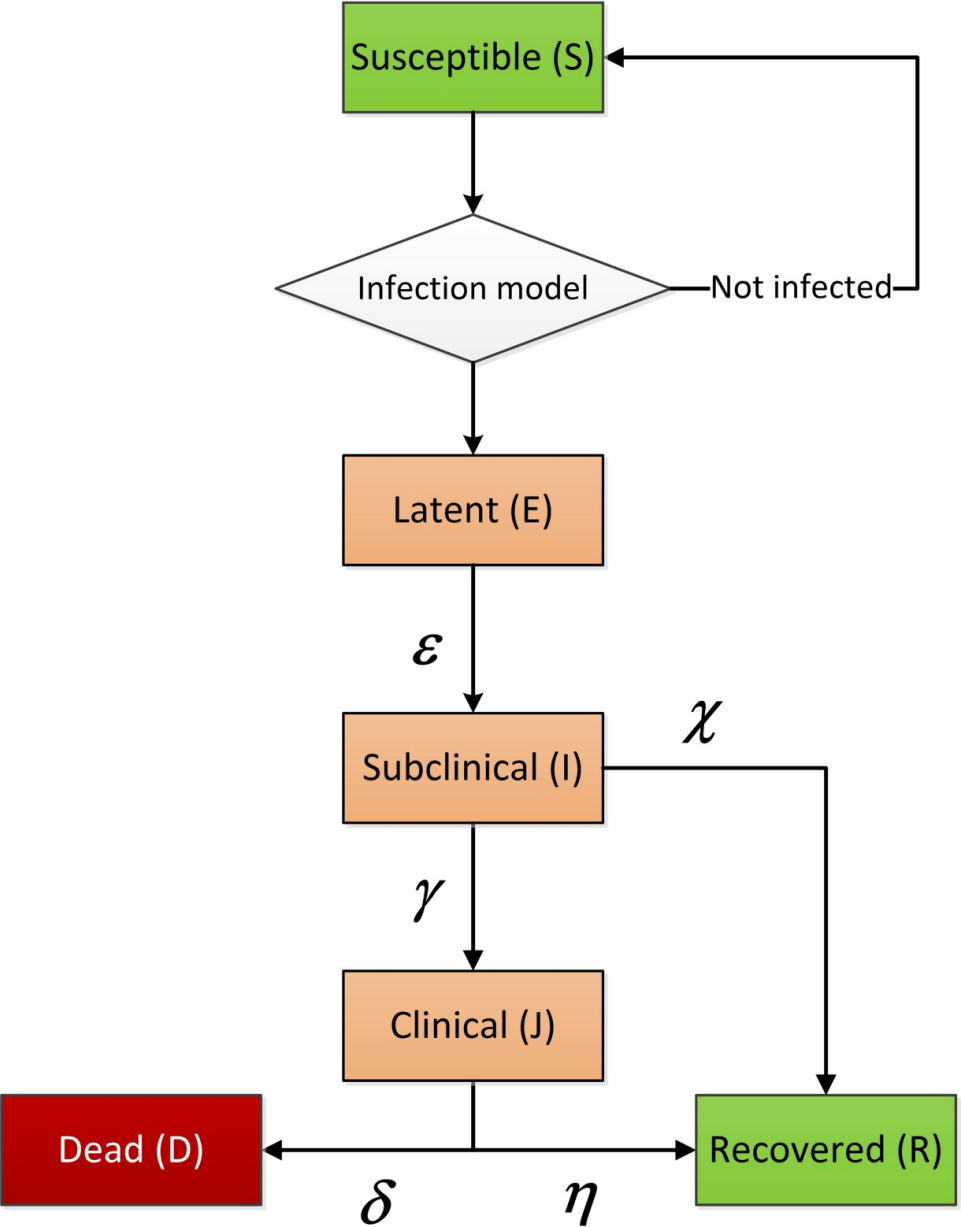
The disease model implemented for FMD. An animal that becomes exposed to FMD is located at the top in the *susceptible* state. The infection model determines if the animal becomes infected and thereby is moved to the *latent* state or if it remains in the *susceptible* state. The animal is thereafter transferred through the model at a stochastic pace and path with probabilities determined by the set of model parameters depicted in the figure.

FMD exhibits a behavior that can be represented by a compartment model. Each animal is transferred between six compartments using discrete time steps. It is a stochastic model implying that each compartment always hold an integer number of animals and a stochastic algorithm is used to determine when animals are transferred. The compartments are *susceptible* (S), *latent* (E), *subclinical* (*I*), *clinical* (J), *recovered* (R), and *dead* (D). Fig 2 shows the structure and flow of the disease model.

#### Model parameters

On the scale of individual animals, there are six model parameters for each species; the susceptibility that determines the risk of becoming infected and five transition parameters that define the transition probabilities between the states for infected animals. Note that parameters for the disease transmission between animals are described in the section Intrafarm disease *spread*. The susceptibilities of the different species for FMDV by the respiratory route has been examined experimentally [40–43]. The quantitative results are troubled by uncertainties but are still informative and useful. Gibson et al. conducted experimental studies on 24 sheep that were exposed to dosage ranging from 4.2 to 1 557 TCID_50_ of the O_1_ BFS 1860 strain of FMDV [41]. Following the definition of French et al. [28], an animal is considered to be subclinically infected (termed *infected* by French et al.) when it has sero-converted or if virus was found in the oesophageal-pharyngeal fluid while the animal is clinically infected (termed *diseased* by French et al.) and it was viraemic or developed lesions. It was concluded that 7 out of 12 sheep exposed to doses below 50 TCID_50_ were infected, while 11 of 12 animals exposed to higher dosage became infected. All infected animals became clinically infected in this study. A similar study was later performed on calves by Donaldson et al. [40] where 18 calves were exposed to a varying amount of the O_1_ BFS 1860 strain of FMDV. In total 16 calves became subclinically infected and 11 became clinically infected. The two uninfected calves were exposed to relatively low dosages, i.e. in the interval 13–25 TCID_50_. Exposure to dosage in this interval also caused infection to three calves. Alexandersen et al. followed up these two studies with an investigation on the susceptibilities for pigs to the O_1_ Lausanne strain [42, 43]. The conclusion was that the MID_50_ values for subclinical and clinical infections lie in the intervals of 300–2 000 and 800–6 000 TCID_50_. We use the average value in these intervals. In the same study they also re-analyzed the experimental data for sheep and cattle described above and gave estimates of the MID_50_ values for subclinical infection for cattle and sheep as 5 and 7 TCID_50_, respectively [42].

These facts suggest that the fraction of subclinical animals that reaches the clinical state is 0.69 for cattle, 0.57 for pigs and 1.0 for sheep. To establish the ratio for pigs, the infection model was utilized with the reported MID_50_-values. That is, the reported MID_50_-dose for subclinical state was used to calculate the susceptibility. Next, by using the reported MID_50_-dose for clinical state and the established susceptibility, the fraction of the pigs that first must have entered the subclinical state was found to be 0.87. This means that 57% of the pigs in subclinical state continue into the clinical state. The total flow of animals out of the subclinical state is divided into two paths, which is described by the parameters *χ* and *γ*. Animals that reach the subclinical state but recovers without entering the clinical state are caught by the *χ*-parameter and vice versa. That is, the fraction of the animals in the subclinical state that directly recovers equals *χ* divided by the sum of *χ* and *γ*. Now, by using this data in combination with the information of the durations of the different disease stages, that is shown in Table 1, all parameters in the disease model, depicted in Fig 2, can be established. It is six parameters for each species and they are presented in Table 2.

**Table 2:** The disease model parameters for all three species.

Parameters	Cattle	Pigs	Sheep
*s* [1/PFU]	0.20	0.00087	0.14
*ε* [1/day]	0.28	0.32	0.21
*χ* [1/day]	0.16	0.19	0.0
*γ* [1/day]	0.35	0.25	0.45
*δ* [1/day]	0.021	0.015	0.045
*η* [1/day]	0.40	0.28	0.86

The transition rates between different disease states are calculated in such a way that the average transition times presented in Table 1 are maintained. PFU is an acronym for *plaque-forming units* and is a common unit of quantity in virology.

### Intrafarm disease spread

**Fig 3:**
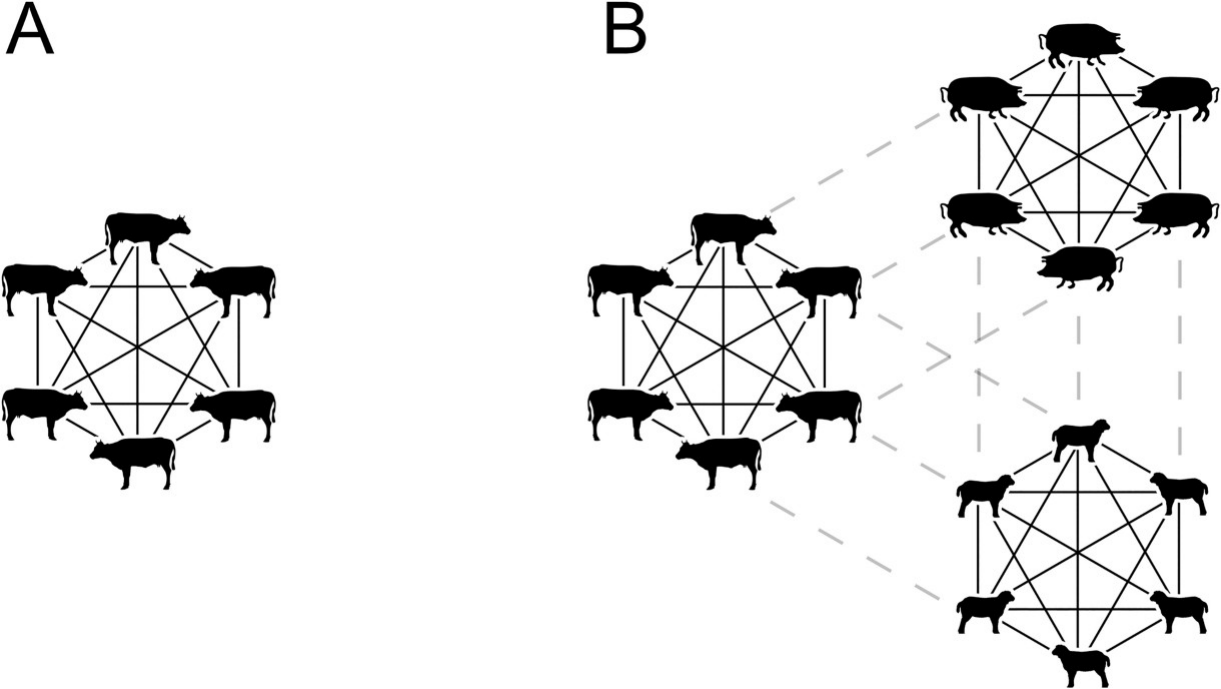
Disease transmission routes within farms. (A) Illustration of a well-mixed group where all animals have equal connection with every other animal in the group. B) Three groups that are all separately well-mixed but have weaker connections to animals of the other groups.

Each individual animal follows the scheme described on the first scale and illustrated in Fig 2. However, the animals are, of course, not isolated but rather located in groups of different sizes on farms. The second scale describes the disease spread within the livestock on a single farm. Animals of the same species are assumed to be well-mixed which means that they have equal connections with all the other animals of that species, see Fig 3A. When a farm have several species, this assumption is still valid within each species. However, there is a heterogeneity present on farms holding several species, as they are often kept separate to some degree from each other. This means that there is a weaker connection between two animals of different species than there is between two animals of the same species, see Fig 3B.

The scales of individual animals and intrafarm disease spread are both modeled simultaneously on the intrafarm layer. The disease model shown in Fig 2 for individual animals can directly be implemented on the farm scale, which means that the farm has six different states with a certain discrete number of animals in each state. Six differential equations, each corresponding to one compartment, describe the dynamic development of the disease on the farm. For each state, a state vector holds the number of animals of each species. The vectors are named *S*, *E*, *I*, *J*, *R* and *D* as marked in the disease model in Fig 2. The order of the species in the vectors have been chosen as [Cattle, Pigs, Sheep] and the first letter is used as index to the state vectors. The equation system is given in Eq (3)

$$\begin{array}{l} \dot{\bar{S}}=-\frac{\beta_{0}}{N}\left(\bar{\bar{\rho }}\bar{\Lambda_{I}}\right)\circ \bar{\Lambda_{S}}\circ \frac{\bar{\Lambda_{F}}}{\Lambda_{F,C}}\\ \dot{\bar{E}}=\frac{\beta_{0}}{N}\left(\bar{\bar{\rho }}\bar{\Lambda_{I}}\right)\circ \bar{\Lambda_{S}}\circ \frac{\bar{\Lambda_{F}}}{\Lambda_{F,C}}-\bar{\varepsilon }\circ \bar{E}\\ \dot{\bar{I}}=\bar{\varepsilon }\circ \bar{E}-\left(\bar{\gamma }+\bar{\chi }\right)\circ \bar{I}\\ \dot{\bar{J}}=\bar{\gamma }\circ \bar{I}-\left(\bar{\delta }+\bar{\eta }\right)\circ \bar{J}\\ \dot{\bar{R}}=\bar{\chi }\circ \bar{I}+\bar{\eta }\circ \bar{J}\\ \dot{\bar{D}}=\bar{\delta }\circ \bar{J} \end{array}$$ (3)

where the operator ∘ is the Hadamard product, i.e. the entrywise product of two vectors or matrices. *N* is the total number of animals on the farm. On this scale, there are likely many different transmission routes present. However, they are in practice impossible to identify and separate. Instead, it is assumed, on the intrafarm layer, that the probabilities for the governing disease transmission routes are all proportional to the airborne excretion rates, which then implies that all transmission routes can be congregated into one transmission parameter. This parameter is *β*_*0*_, which thus represents the probability for exhaled pathogens to be present in the inhalation volume of the other animals on the farm. *β*_*0*_ can also be considered a scaling factor for intrafarm disease transmission between infectious and susceptible animals.

${\bar{\Lambda }}_{S}$ describes the total susceptibilities of the animals, i.e. the risk of an animal to transit into the latent state when exposed, where *s* is the susceptibilities.

$${\bar{\Lambda }}_{S}\equiv \left[\begin{array}{c} s_{C}S_{C}\\ s_{P}S_{P}\\ s_{S}S_{S} \end{array}\right]$$ (4)

${\bar{\Lambda }}_{I}$ describes the total infectiousness of the animals and is given in the number of emitted PFU per day from each species, where *i* and *j* are the excretion rates at the states *I* and *J*.

$${\bar{\Lambda }}_{I}\equiv \left[\begin{array}{c} i_{C}I_{C}+j_{C}J_{C}\\ i_{P}I_{P}+j_{P}J_{P}\\ i_{S}I_{S}+j_{S}J_{S} \end{array}\right]$$ (5)

$\bar{\Lambda_{F}}$ describes the inhalation rate in cubic meters per day and Λ_*F*,*C*_ is the inhalation rate for cattle. The fraction between $\bar{\Lambda_{F}}$ and Λ_*F*,*C*_ is used in Eq (3), i.e. the relative inhalation rate. The relative inhalation rate is used to keep the scaling parameter *β*_*0*_ dimensionless.

$$\bar{\Lambda_{F}}\equiv \left[\begin{array}{c} \Lambda_{F,C}\\ \Lambda_{F,P}\\ \Lambda_{F,S} \end{array}\right]$$ (6)

The matrix $\bar{\bar{\rho }}$ is an assortative-factor that describes the mixing of the three species of animals, illustrated in Fig 3B. The matrix is symmetric, i.e. *ρ*_*ab*_ = *ρ*_*ab*_. Furthermore, it is assumed that all three species mix equally on the farm and we set all off-diagonal elements to *ρ*_*0*_.

$$\bar{\bar{\rho }}=\left[\begin{array}{ccc} \rho_{CC} & \rho_{0} & \rho_{0}\\ \rho_{0} & \rho_{PP} & \rho_{0}\\ \rho_{0} & \rho_{0} & \rho_{SS} \end{array}\right]$$ (7)

Assuming that the total number of contacts are kept constant regardless of the grade of inter-species mixing, we get the condition

$$\rho_{aa}=\frac{1}{N_{a}}\left(N-\rho_{0}\left(N-N_{a}\right)\right)$$ (8)

for the diagonal elements for each species *a*. If *ρ*_*0*_ = 1, the mixing is completely random while a value below one indicates a certain degree of segregation between the animal species. A value above one would imply that two animals are less likely to have contact with each other if they were of the same species than if they were of different species. This is an unrealistic scenario, and Eq (9) is actually only valid for *ρ*_*0*_*≤1*. The assortative-factor *ρ* is difficult to assess properly. However, it is clear that the livestock in Sweden has a positive assortative nature implying a value of *ρ*_*0*_<1. We use a value of 0.5 which is in line with the estimated value for livestock in England during the FMD outbreak in 2001 [44].

The vectors $\bar{\varepsilon }$, $\bar{\gamma }$, $\bar{\chi }$, $\bar{\delta }$, and $\bar{\eta }$ describe the transition rates for the species from one state into another state according to Fig 2. The vectors hold the reciprocal of the expected time length in each stage for cattle, pigs, and sheep, respectively, and they are presented in Table 2.

The infectiousness of the farm as a whole is directly described as

$$Z=\sum_{q=1}^{3}{\bar{\Lambda }}_{I}\left(q\right)$$ (9)

where *q* is the index of the species. *Z* serves as source term for the long-range disease spread from the farm on the interfarm layer and has the unit of released plaque-forming units (PFU) per day for the entire farm. This entity is used for interfarm disease spread modelling.

#### Model parameters

Four new parameters are added to the system for the intrafarm disease spread model. Two of them refer to the virus excretion from animals in the subclinical and clinical states. The third parameter quantify the inhalation volume per days for each species and the fourth parameter, *β*_*0*_, is the scaling parameter for the overall within farm transmission probability of FMD and is not species specific.

The excretion of airborne FMD virus has been examined by Alexandersen et al. [43, 45, 46] and their results are used in this study. The excretion rate varies over time with peak values of 10^4.3^ TCID_50_ per day per animal for cattle and sheep. Values of 10^5.8^, 10^6.1^ and 10^6.4^ TCID_50_ per day and per pig are reported depending on strain [43]. We have chosen to use the middle value of 10^6.1^ TCID_50_ per day and pig in this study. In a well-mixed compartment model, the animals in the same state have the same properties, which means that the rates must be constant over time for each compartment. Representative mean values in both subclinical and clinical states are estimated by inspection of time varying data for sheep in [45] and for cattle and pigs in [46]. The subclinical and clinical values for sheep were found to be approximately 80% and 50% of the peak value while the corresponding values for cattle are 30% and 50% and for pigs 20% and 80%. The results, converted to PFU, are presented in Table 3.

**Table 3:** The model parameters used in this study for the intrafarm disease spread of FMD.

Parameters		Cattle	Pigs	Sheep
*i*	[PFU/day]	4 100	170 000	11 000
*j*	[PFU/day]	6 900	690 000	6 900
Λ_*F*_	[m^3^/day]	150	50	15
*β*_*0*_	[au]	0.03	0.03	0.03

The parameters differ significantly between the species. Pigs have a significantly higher excrete rate than both cattle and sheep.

**Fig 4:**
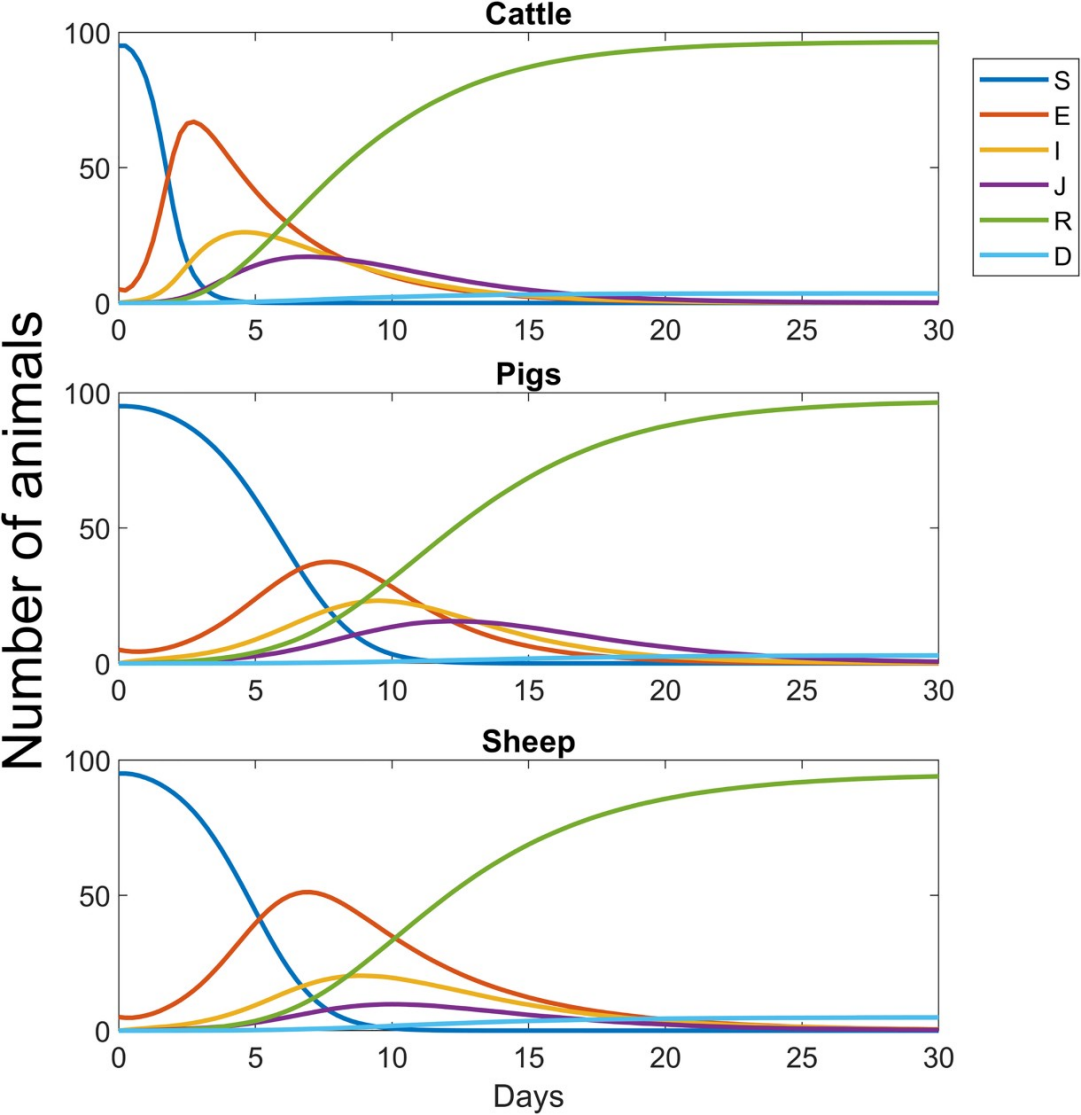
The development of FMD amongst the animals on an infected farm, i.e. intrafarm disease spread. The disease is initiated with 5 infected and 95 uninfected animals in all cases. The disease develop quickest in the case with cattle and slowest for pigs.

The scaling parameter *β*_*0*_ has not explicitly been determined in any study. However, by comparing the intrafarm model with observation data it can be estimated. The purpose of the parameter is to scale the probability of disease transmission on the intrafarm layer. FMD is known to spread widely on infected farms, which means that *β*_*0*_ is required to exceed some lower limit value. For all values above this, the role of *β*_*0*_ is mainly manifested by the pace of the spread. At any time, the probability of airborne disease transmission to neighboring farms depends on the total excretion of pathogens from all animals on the infected farm, which means that the speed of a chain of interfarm infection can be used to estimate the development of the disease at infected farms. Sellers et al. presented a detailed study of the spread of FMD that took place in Northumberland, UK, in July-September 1966 [24]. Of particular interest is the Yetlington case where a series of airborne spreads between farms could be identified. The majority of the farms investigated held cattle with a smaller fraction of sheep and pigs. No pigs were infected at any farm though. By means of disease reports and meteorological analyses, routes and times of transmissions were suggested. Fourteen farms were infected during five weeks and possibly up to seven farms acted as sources. The network of disease transmission included up to three subsequent interfarm transmissions that spanned from 3–16 days each. Most transmissions occurred between 7–11 days after the farms had been infected. In a similar investigation into the Hampshire epizootic 1967 the corresponding time interval was 4–9 days [47]. In addition, the report on the Northumberland case provides the number of affected animals in combination with the date of disease reports and the estimated first date of clinic disease on each farm. Altogether, this set of information acts as an indicator of the transmission rate of the disease at an infected farm. Now, it is reasonable to assume that most historically documented interfarm disease transmissions occurred before the peak infectiousness was reached since culling typically is conducted shortly after the disease is discovered. This hypothesis is also supported by reports on culling taking place when only few animals were found infected [24]. The intrafarm model was compared to this set of information and the value *β*_*0*_ = 0.03 was found to provide the desired temporal rate of the intrafarm spread. Using this value with simulation time step of 6 h, farms hosting 100 animals of either cattle, pigs and sheep have peak infectious values at 9, 16, 14 days, respectively, when a single animal was initially infected. Initializing the simulation with five infected animal decreased these numbers to 6, 12 and 10 days. Fig 4 shows the temporal development of the disease on a single infected farm that holds either only cattle, only pigs or only sheep. It is in particular clear that the disease develop slower for the case with a pig farm than for the other two species, especially cattle.

These time intervals are also in agreement with the within-farm model of Chis Ster et al. [48], which is based on the 2001 UK epizootic. Note that the overall epizootic spread in absence of culling is not sensitive to this parameter since it mainly regulates the speed of intrafarm disease spread and, within a reasonable interval, only have a minor impact on the magnitude of the epizootic scale.

Finally, Donaldson and Alexandersen report average inhalation rates of 150, 50 and 15 m^3^/24 h for Cattle, Pigs and Sheep respectively [49].

### Interfarm disease spread

So far, we have covered the disease model for individual animals and the disease spread within an infected farm (i.e. a farm where at least one animal is infected). The top layer in Fig 1 depicts schematically the disease spread from infected farms to non-infected farms, a process that is modelled by a transmission network.

The interfarm disease spread is in this study modelled as a network of farms with anisotropic transmission probabilities between them. The utilization of network representation for disease spread between farms is not novel. For instance, Webb used a network description of farms holding sheep in Britain [50]. This network modelled both disease transmission between closely located farms and disease spread due to animal transportations to common grounds. Further, a network analysis of the UK 2001 epizootic was conducted by Shirley and Rushton [51]. A comprehensive review on the field of epidemiology on networks is presented by Danon et al. [52]. Many investigations on spatial networks use transmission kernels that describe the probability for disease transmission between farms. It is somewhat unclear what processes are included in such modelling procedures, but all causes for local disease spread is often congregated into one common parametrized term without further details. It is noteworthy that the kernels typically depend on the Euclidean distance [44, 53–55], which implies that the system is assumed to be isotropic. This simplification is not used in this study.

#### Transmission probability

Let *p*_*ab*_(*t*,*t’*) be the transport probability density for one unit of the pathogen (PFU) from farm *a* at time *t’* to the farm *b* at time *t*. The inhalation probability of the present airborne pathogens is a linear function of the concentration, depending only on the inhalation rate of the animal. This means that the probability for one specific animal *k* at farm *b* to inhale that pathogen during the short time interval [*t*, *t*+Δ*t*] is *p*_*ab*_(*t*,*t’*)Λ_*F*_Δ*t*. The expected total number of inhaled pathogens, originating from farm *a*, for a specific animal *A*_*b*,*k*_ at farm *b* during the time interval [*t*, *t*+Δ*t*] is *n*_*k*_.

$$n_{k}=\Lambda_{F,k}\Delta t\int_{-\infty }^{t}p_{ab}(t,t^{\prime })Z_{a}\left(t^{\prime }\right)dt^{\prime }$$ (10)

*Z*_*a*_ describes the total number of pathogens that the infected animals at farm *a* exhale per day. The atmospheric dispersion process takes place on a shorter time scale than the animal disease model and *Z* can therefore be considered constant during the transport period of a pathogen between farms, i.e. *Z*(*t’*) = *Z*(*t*) when *p*_*ab*_(*t*,*t’*) > 0. Further, *p*_*ab*_ only depends on the time that has elapsed since the pathogen was exhaled, and by introducing the parameter *τ* ≡ *t*-*t’* the time integral can be rewritten in a form that is easier to interpret.

$$n_{k}=\Lambda_{F,k}\Delta tZ_{a}\left(t\right)\int_{0}^{\infty }p_{ab}(\tau^{\prime })d\tau^{\prime }.$$ (11)

The time integral in Eq (11) describes the likelihood for pathogens to travel from farm *a* to farm *b* and we define the parameter *W*_*ab*_ as

$$W_{ab}\equiv \int_{0}^{\infty }p_{ab}(\tau^{\prime })d\tau^{\prime }$$ (12)

which is referred to as the *connection strength*. The connection strength is the probability density that a virus particle released at farm *a* will be transported to farm *b* and has the unit day·m^-3^. Now, this integral can be identified as the time integrated concentration field with a unit source, called the *exposure field*, which can be directly obtained from dispersion models. To generalize, the vector of expected inhaled viruses for all animal types is

$$\bar{n}=\bar{\Lambda_{F}}\Delta tZ_{a}\left(t\right)W_{ab}.$$ (13)

#### Farm infection

Consider an uninfected farm that is exposed to the disease from a neighboring farm that is infected. All animals of each species have equal probabilities to become infected during the time step Δ*t*. These probabilities, $\bar{P_{inf}}\left(\bar{n}\right)$, are given by Eq (2) with the number of inhaled pathogens provided by Eq (13). The total number of animals that become infected, $\Delta \bar{E}$, during the time step Δ*t* is a stochastic variable that follow the binomial distribution, *B*, for each species separately.

$$\Delta \bar{E}=B\left(\bar{S},\bar{P_{inf}}\left(\bar{n}\right)\right)$$ (14)

The linear estimate of the probabilities is a more straightforward method, i.e.

$$p\left(\Delta \bar{E}\right)=\bar{P_{inf}}\left(\bar{n}\right)\circ \bar{S}$$ (15)

which provides the same infection rate in most occasions, especially for low probabilities. Indeed, the expectation values are actually identical. However, there is one difference to consider. The probability of disease spread within an infected farm is significantly higher than that between farms. A single infected animal is most likely enough to infect the entire farm. That is, the difference between infecting zero and one animal is much more influential than the difference between infecting one and two animals. Since the binomial distribution captures the entire distribution, it provides accurate results and it was therefore implemented. Due to efficiency reasons the linear approximation was still used as standard but replaced with the binomial method when $\max\left(p\left(\Delta \bar{E}\right)\right)>0.25$ which resulted in a fast and, to a high degree, accurate algorithm.

Eqs (2), (13) and (14) are the governing equations for interfarm disease transmission in a network of farms. The missing component so far is the connection strengths between the farms, which is addressed next.

#### Atmospheric dispersion modelling

Atmospheric dispersion simulations were deployed to quantify the connection strengths *W*_*ab*_ between all farms in the network. As discussed above, the connection strength is the probability density that a pathogen released from farm a reaches farm *b*, and is given in Eq (12). This entity can be found by numerical simulation using a dispersion model for specific meteorological conditions. The model LPELLO developed by the Swedish Defence Research Agency, was used here for this purpose. LPELLO is a Lagrangian particle model based on the Langevin equation [56, 57]. Turbulence is modelled by means of a Wiener process where the atmospheric turbulence determines the magnitude of the stochastic contribution. The output from a simulation is an exposure field, Ψ, which is the same as the dose field using a unit source. This field has the unit of day·m^-3^ and equals the time integral of the concentration normalized by the total number of released pathogens.

$$\Psi \left(\bar{r}\right)=\frac{1}{M}\int_{0}^{\infty }C\left(\bar{r},t\right)dt$$ (16)

where *C* is the concentration and *M* is the total released number of pathogens, i.e. *M*≡∬*C dtdV*. The exposure field, with farm *a* at the origin and $\bar{r}$ set to the position of farm *b*, equals the connection strength *W*_*ab*_ for the particular meteorological conditions used. However, the variations in the meteorological conditions must be considered which implies that a more extensive method is required. The most important input data for the atmospheric dispersion simulations is described here, followed by a description of the data processing required to obtain the desired connection strengths.

A statistical approach is utilized in this study, meaning that historically data is used to set up distributions of measured wind directions and wind speeds. The preconditions for atmospheric transmission depend on several meteorological properties in a nonlinear manner. This means that estimating a connection strength using a single simulation based on the mean values of all meteorological data is a crude simplification. Instead, the connection strength is better estimated by assessing transmission probabilities for the distribution of preconditions, and thereafter calculating the expectation values. Based on daily and seasonal variations in meteorology, the empirical data was divided into eight different meteorological groups: four seasons in combination with daytime or nighttime. Sweden is located in the temperate zone where the seasonal variations are evident. The fastest changes take place close to the equinoxes. To account for this, the seasons were defined as November-February (*winter*), Mars-April (*spring*), May-August (*summer*) and September-October (*autumn*). Note that spring and autumn only occupy two months each while winter and summer occupy four months each. The meteorological conditions during each season are assumed statistically invariable. The time-period of an epizootic is typically shorter than the seasons, which means that simulations of an outbreak can be carried out by applying statistics from one season solely. The daily variations were captured by dividing the meteorological data in two groups: 08:00–20:00 as *daytime* and 20:00–08:00 as *nighttime*. The exposure fields for daytime and nighttime will differ, but the resulting connection strength is calculated by the mean of these fields as will be discussed in more detail later. By using this statistical approach, the connection strengths are time independent during a season. Note that the link between two farms is asymmetrical, meaning that *W*_*ab*_≠*W*_*ba*_.

Hugh-Jones et al. conducted a statistical investigation of the correlation between meteorological conditions and the rate of airborne spread of FMD [18]. They observed a correlation between the disease spread and the wind direction, while significantly weaker correlation was detected between disease spread and precipitation. The former finding is expected and the latter suggests that we may disregard precipitation in the atmospheric dispersion modeling for these short distances. The required meteorological input data is therefore limited to wind speed and the coupled wind direction for the eight different meteorological groups defined above. Since these distributions are highly anisotropic over the country, such datasets are desired with a spatially distribution. Additional information needed to conduct atmospheric dispersion simulations could be inferred from these meteorological data sets, which is discussed below.

**Fig 5:**
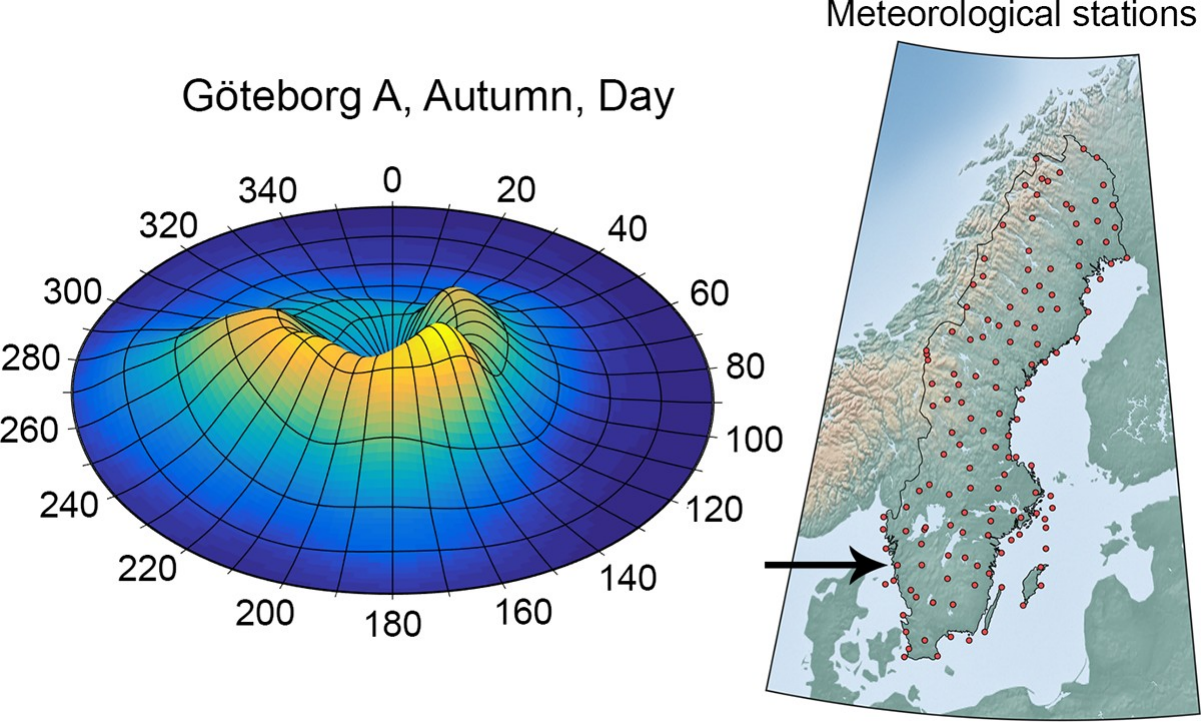
The meteorological preconditions are represented by local wind roses. (A) One of the wind roses for the station *Göteborg A* plotted with polar coordinates. Wind speed and wind direction are represented by the radius and the angles, respectively. High values implies a high probability for that combination of wind speed and wind direction. Since the wind rose constitutes a probability distribution, the volume below the surface equals one. The figure clearly shows that the meteorological preconditions are anisotropic. (B) The 138 automatic meteorological stations of Sweden. Statistic datasets were gathered for each such station, together providing a proper description of the meteorological conditions of Sweden. Panel B uses a map from Natural Earth (public domain).

Comprehensive meteorology data was required to be able to conduct this analysis and obtain the connection strengths. The Swedish Meteorological and Hydrological Institute, SMHI, provides an open database containing a vast amount of historical weather observations at a multitude of weather stations spread around Sweden [58]. Using the open database [59], historical data can be downloaded for many parameters using a map interface with all the meteorological stations. The wind speed and wind directions are of interest for this study. Spreadsheets with time resolved data for these two parameters are readily available for manual download directly from the database. Data has been collected systematically since 1939. However, there are discontinuities in the dataset; stations have been added, removed or relocated during this extensive period. SMHI has reviewed the dataset and labelled the measurements according to the reliability thereof. Each line in the spreadsheets is marked with a quality flag. To maintain high quality in the statistics, we have only used lines marked with “G” which means the values are approved. Further, following recommendations from SMHI, only the set of automatic meteorological stations were used (labelled with an “A” after the station name). This subsystem, which is shown in Fig 5, is composed of 138 stations that was constructed and taken into operational use mainly in the nineties. The spatial distribution of the stations allowed for a good description of the regional weather. All stations measure wind directions and wind speed at 10 meters height with an hourly average. The data was quantitatively examined which revealed that one station, *Huvudskär Ost A*, offered significantly less data points that the others and possibly too few to provide reliable statistics. Fortunately, this station is located far out in the Baltic Sea and thus it did not influence the outcome of this study. The meteorological data set amounts to a total of 12.2 million measurement points used here to describe the weather conditions in Sweden statistically.

An efficient presentation method of the wind distribution is to use wind roses. Wind roses are traditionally used to show distribution of wind velocities using different discrete sections of wind directions. The wind roses in this study instead constitute two-dimensional continuous bivariate probability distributions over direction and speed; an example of a wind rose is showed in Fig 5A. The distribution was assembled using two-dimensional kernel density estimators of the point data. Gaussian kernels were used with standard deviations of 15 degrees for the wind direction and 0.75 ms^-1^ for the wind speed. Special care is required for windless measurements since the wind direction carry no information in this case. This has been solved by attributing such measurement equally to all wind directions. The procedure described here resulted in eight different wind roses, one for each meteorological group, for each station. The wind roses were normalized which means that they may act as probability density functions for the wind speed and wind direction.

In the next step, wind roses at the locations of all farms were constructed by means of spatial interpolation using the wind roses corresponding to the meteorological stations. Let us label the wind roses *ζ*, the spatial position of meteorological station *k* in a network of *K* stations as ${\bar{r}}_{k}$, the *distance weight* as *ω*_*ak*_, and the *station weight* as *α*_*k*_. The wind rose at position ${\bar{r}}_{a}$ may then be spatially interpolated as

$$\zeta \left({\bar{r}}_{a}\right)=\frac{1}{\omega_{ak,tot}}\sum_{k=1}^{K}\alpha_{k}\omega_{ak}\left({\bar{r}}_{k},{\bar{r}}_{a}\right)\zeta \left({\bar{r}}_{k}\right).$$ (17)

This distance weight is a function that decrease with distance to represent the decline of correlation. The distance weights are normalized which is obtained by the inclusion of the factor

$$\omega_{ak,tot}\equiv \sum_{k=1}^{K}\omega_{ak}\left({\bar{r}}_{k},{\bar{r}}_{a}\right).$$ (18)

If *ω* is independent of *ζ* it can be described as linear are therefore known a priori. The distance weights depend on the distance but also on the direction between the positions. Sasaki [60] and Barnes [61] suggested a Gaussian function for the decline in weight, see Eq (20).

$$\omega_{ak}\left({\bar{r}}_{k},{\bar{r}}_{a}\right)\propto e^{-\frac{{\left(\left|{\bar{r}}_{a}-{\bar{r}}_{k}\right|\right)}^{2}}{2R_{ak}^{2}}}$$ (19)

*R*_*ak*_ is a range parameter deciding the rate of decline with distance. The decline in spatial correlation may be anisotropic due to large-scale variations of the topography or type of landscape. The most prominent features causing a short correlation in the case of wind is the transition between sea and inland and the transition from flat land to mountains. Angle dependent values of *R*_*ak*_ was manually asserted by inspection of the local topography and coastlines for each meteorological station. Measurements from different stations are assumed uncorrelated in the sense that the specific positions of the stations are not considered. Such an assumption could lead to an exaggerated impact of measurements from different stations positioned in close proximity to each other. This is in general not the case for the Swedish meteorological station network. There are two exceptions though where two and three stations, respectively, are located close to each other. The stations weights, α_*k*_, are implemented to resolve this issue. They are set to unity for all stations except these five stations where the same weight has been divided intermutually. In summary, each farm was asserted a wind rose for each of the eight meteorological groups. These wind roses were then utilized in the atmospheric dispersion simulations.

An important factor for the airborne transport of aerosols, and thereby the connection strengths, is the atmospheric stability. It is governed by the vertical temperature gradient and it determines the turbulent mixing of the air. The stability varies locally with both time and position and it depends on several meteorological conditions. Atmospheric stability is often categorized into discrete Pasquill classes [62]. There are six different classes labelled A-F where the first three, i.e. A-C, correspond to an unstable atmosphere (convective boundary layer), D means neutral atmosphere, and E-F stable atmosphere. The wind roses represent the probability of convective winds between farms. However, corresponding distributions of atmospheric stabilities are required to be able to couple this information, by means of dispersion simulations, to quantitative connection strengths. An algorithm was constructed that estimates the most representative atmospheric stability by using the season, time of the day (daytime or nighttime), the 10 m wind velocity, cloud and snow coverage and the latitudinal position as input data.

The solar altitude is an important factor since it is the dominating factor for the incoming radiation. It can be calculated from the latitudinal position, season and time of the day. High solar altitude in combination with low wind speed is associated with unstable conditions (A, B or C). In contrast, low solar altitude and low wind speed is associated with stable conditions (E, F) while high wind speed is often found during neutral stability conditions (D).

Cloud cover influences the radiation balance. Incoming short-wave radiation is partly reflected by clouds while long-wave radiation becomes partly re-emitted back to the ground. Presence of clouds therefore results in lower probability of the extreme stability classes (A, F). Cloudiness is commonly measured in integer number of eights. It is assumed that a constant value representing the average cloudiness is applicable in this context. One might argue that 4/8 should be suitable. However, there is a threshold at this value in the specific algorithm used, which implies that the neutral stability would become overrepresented. A constant value for the cloudiness of 3/8 was therefore chosen which provides better estimations of the stabilities for the different conditions set up by the other input parameters.

Snow coverage is of interest since it influences the radiation balance, which in turn influences the atmospheric stability. There is a significant difference in snow coverage over Sweden; the northernmost part is completely snow free only during summer while the southernmost part only occasionally experiences snow even during winter. In this algorithm, Sweden is divided into four geographical regions based on climatology. The expected degree of snow coverage at a location is estimated from the present season in combination with its geographical region.

**Fig 6:**
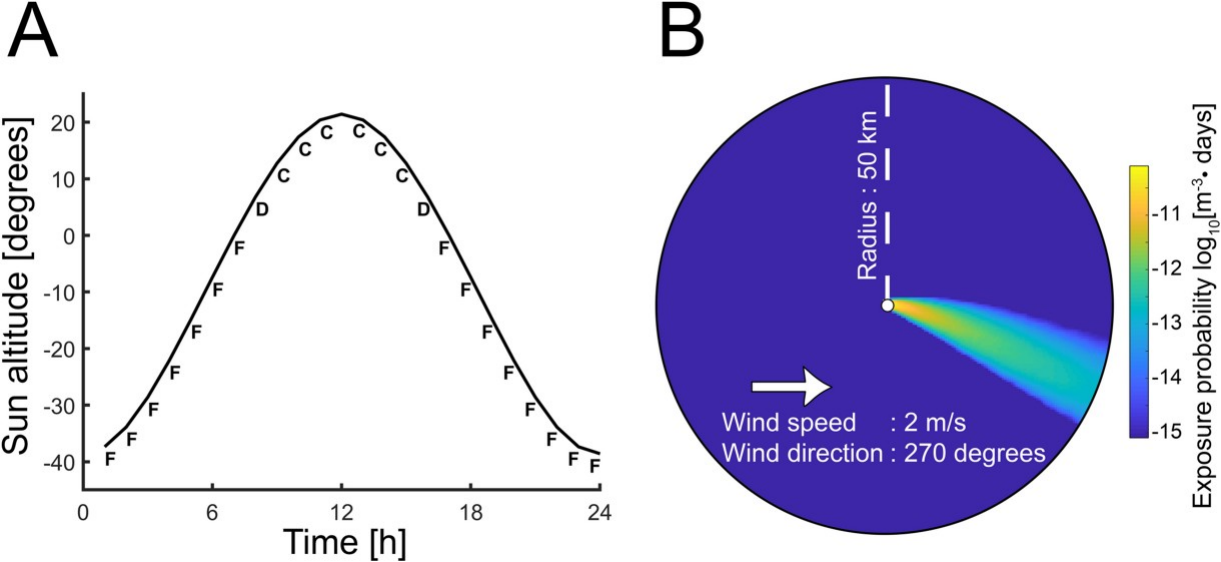
The atmospheric stability influences the atmospheric transmission. (A) The atmospheric stability changes over the day due to the influx of heat from the sun. (B) The exposure field following an atmospheric dispersion simulation, i.e. the exposure probability density, with a source at origin and a westerly wind with a speed of 2 ms^-1^ (at the height of 10 m) in the stability class E. The off-plume value is zero and is here set to 10^-15^ day m^-3^ for visualization reasons only.

The algorithm can be described briefly as follows. The solar height, cloud and snow coverage provide a radiation index. Based on the wind velocity, the stability is calculated from a table look-up. One stability is calculated for each hour in the provided time of the day (daytime or nighttime) and an average stability that is considered representative for the dispersion process during this time interval is obtained. The algorithm is able to capture the predominate physics of the atmospheric stability. Fig 6A shows how the stability varies during the day for one specific set of prerequisites. The transition periods between different stabilities are short since the sun altitude have a stepwise behavior on the produced result. It is noteworthy that the stability in general varies strongly with the associated wind speed, i.e. the radius in the wind roses, which affects the dispersion simulations and the connection strengths.

Another important property of the atmosphere is the planetary boundary layer (PBL) height since it determines the mixing height. In the convective boundary layer (stability classes A to C), the PBL height is set to 1 000 m. This is a simplification and only represents the well-developed convective boundary layer. During transition from stable conditions to a convective boundary layer other values, usually lower, are obtained. In the stable case (stability classes E and F), the PBL height is obtained implicitly by applying similarity theory for the boundary layer and using the stability, wind speed and roughness as constraints. A roughness parameter of 0.2 m was used consistently. From the stability and the roughness, the proper interval of the Monin-Obukov length is determined using the Golder diagram [63]. In a similar approach, the proper interval of the friction velocity is calculated using the Monin-Obukov lengths and wind speed. Thus, estimates of the Monin-Obukov length and the friction velocity are set by taking the average values of these intervals. From these parameter settings, the PBL height can be calculated by analytically solving a second-degree polynomial [64]. In the neutral case (stability class D), there is no well-defined interval of the Monin-Obukov lengths, we therefore set a representative value by expert judgement (L = 5 000 m). From this value, the friction velocity and the PBL height can be calculated by solving the same second-degree polynomial as for the stable case.

Given the PBL height and the wind speed, the altitude depending wind speed profile is computed within the PBL applying similarity theory according to [65] for stable and neutral boundary layers and according to [66] for convective boundary layers. As a final step, a consistency check between the variables are performed and we allow the algorithm to slightly modify the friction velocity to have a representative set of parameter values with respect to stability, wind speed, roughness, Monin-Obukov length, friction velocity and PBL height.

An important feature for atmospheric dispersion is aerosol dynamics. This concept may include many components of which virus survival rate during transport and aerosol deposition are of primary interest here. The survival rate of aerosols is dependent on the temperature but most importantly on the humidity. It has been reported that a relative humidity above 55% provides beneficial conditions such that the virus survival rate can be described as *e*^-*λt*^ while the viruses are unable to spread efficiently at lower humidity [67]. Reports from SMHI show that humidity in Sweden is rarely below 55% [68]. In fact, the humidity is mainly above 70% why the exponential decay function was applied for the viability of the airborne viruses. Experimental data has shown that the half-life time of FMD viruses is 2 hours or more [67]. A decay value corresponding to 2 hours has been used previously in modeling of FMD [7, 69] and we followed these examples implying a decay parameter of λ ≈ 10^-4^s^-1^. The virus survives best under cool temperature and high humidity. It has been shown that under normal meteorological conditions in Sweden, the temperature will have negligible influence and this environmental factor is therefore ignored in this study [70].

The FMD-virus spreads using water based aerosols as carriers. The deposition during atmospheric transmission of aerosols depends on the size of the aerosols wherefore knowledge regarding the size distribution for the exhaled aerosols is required. In 1969 Sellers et al. reported a distribution of the aerosol diameter with 65–71% larger than 6 μm, 19–24% between 3 and 6 μm and 10–11% smaller than 3 μm for pigs [71], and corresponding values of 50%, 25% and 25% for cattle [72]. The data for cattle was reported to have higher uncertainties than the data for pigs. Revisiting the topic of aerosol size distribution, Gloster et al. found in 2007 that the particle sizes of aerosols released from infected pigs were log-normally distributed with evenly distribution between the three size intervals mentioned above [73]. The discrepancy between the distributions shows that there might be an unresolved uncertainty present in this distribution. Using the findings of Gloster et al., the particle diameter distribution used had a log-normal distribution with mean and standard deviation of the logarithmic values of log(4.2 μm) and 0.8, respectively. Large droplets experience a higher settling velocity and therefore an increased deposition rate. Dry deposition was utilized in this study with a deposition velocity of 10^−3^ ms^-1^ for the water based aerosols [74]. Furthermore, we assume that the pathogens are homogeneously distributed in the excreted aerosols, which implies that the number of viruses scales cubically with the aerosol diameter.

Finally, experience from epizootic outbreaks has shown that airborne transmission often occurs on distances below 10 km but spread over significantly larger distances have occasionally been confirmed. We have introduced a cut-off at 50 km implying that we assume that spread over longer distances than that have negligible influence and is negligible. This limit is used for numerical purposes only since it reduces the number of weak connections in the transmission network significantly.

A connection strength cannot be calculated by one single simulation since there are variations in the preconditions as discussed. To address this, a number of pre-calculated exposure fields for wind speeds ranging from 1 to 14 ms^-1^, with steps of 1 ms^-1^, was first calculated for each stability class. These pre-calculated exposure fields were established by means of simulations using all the methods and properties described above. Fig 6B shows the exposure field for the case with Pasquill stability class E and a wind speed of 2 ms^-1^ for a certain wind direction. The exposure fields for different wind directions are readily obtained by a rotation of this field. However, exposure fields corresponding to other stabilities and/or wind speeds need to be numerically calculated separately by means of atmospheric dispersion modelling. These fields then served as reference data in an interpolation process to obtain the desired exposure fields for different distributions of meteorological conditions. The connection strengths were quantified as follows.

The wind roses for two farms were combined to yield a single wind rose that represents the meteorological conditions for the wind speed and wind direction governing the airborne transport between the two farms. For any position in the wind rose, the wind speed and wind direction is obtained. With this wind speed and information of the geographic location of the farms, season and time of the day, the atmospheric stability was inferred using the algorithm described above. An exposure field, corresponding to that position in the wind rose, was then interpolated by utilizing the set of pre-calculated exposure fields. The exposure field was rotated according to the wind direction in combination with the geographic positions of the farms, yielding the exposure value corresponding to the position in the wind rose was obtained. This exposure value was then weighted by the probability that this particular combination of speed and direction occurs, i.e. the value for the corresponding position in the wind rose multiplied with the area segment of the wind rose represented by that position. As mentioned earlier, the wind roses were normalized and are to be considered as bivariate probability density functions. This procedure was conducted using the datasets for both daytime and nighttime whereby the mean value was calculated. The obtained value is a measure of the transport probability between the farms for these conditions. This entire procedure was then repeated with steps of 2.5 degrees for the wind direction and 0.25 ms^-1^ for the wind speed over the entire wind rose. When all sample combinations were summarized, the desired connection strength was finally obtained. Note that this connection strength is valid for one season. This algorithm was therefore conducted for each season. The complete network for interfarm disease spread was constructed by calculating the connection strengths for all combinations of farms for all seasons.

### Livestock data

**Fig 7:**
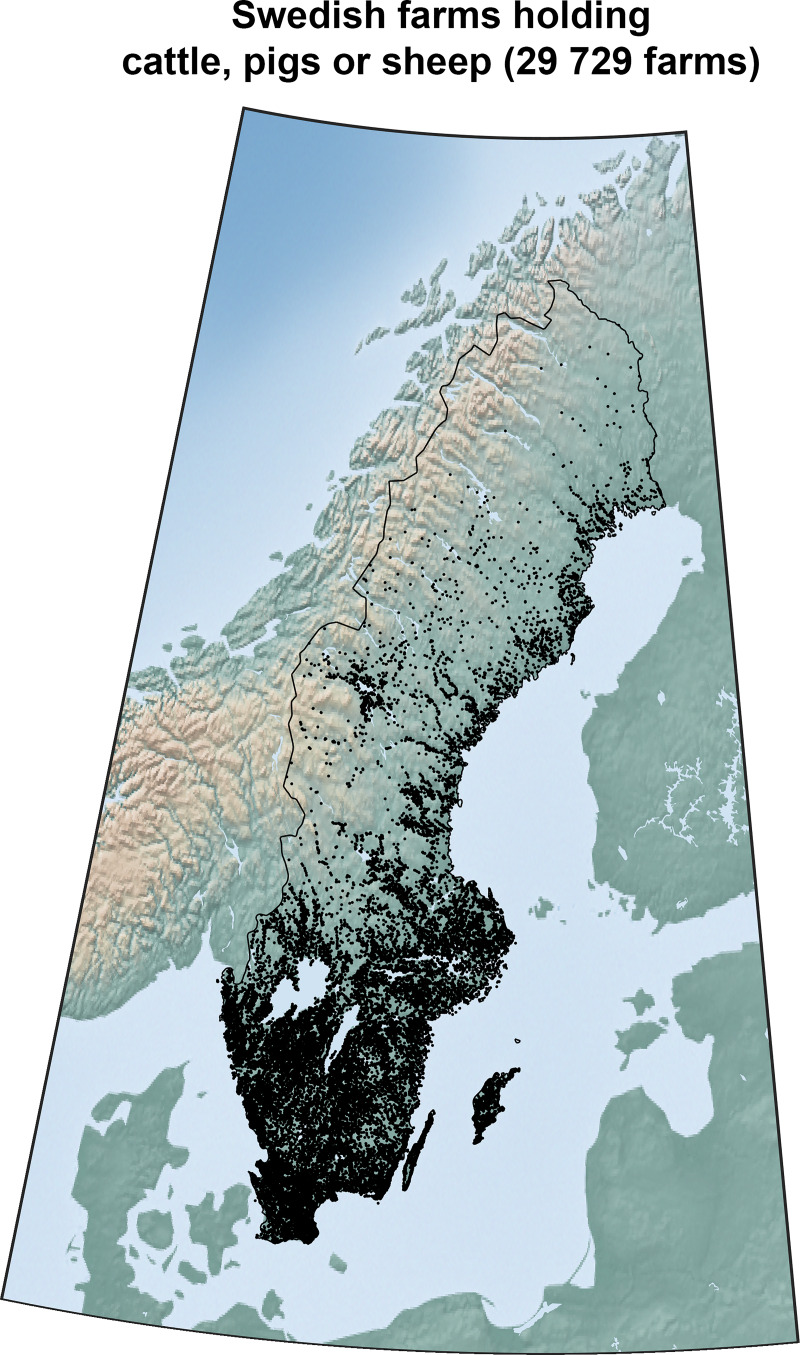
The farms in Sweden holding any of the three species cattle, pigs or sheep. There is a significant dominance of farms in the southern part of Sweden while the inland of the north harbors very few farms due to low population and disadvantages preconditions. The map originates from Natural Earth (public domain).

The data describing the locations of the farms and the quantity of their livestock was extracted from the database holding the official information reported to the Swedish Board of Agriculture (SJV) from farmers, breeders and abattoirs. The locations are provided by geographic coordinates for each PPN (acronym for the Swedish term for “production location number”) which is the administrative equivalence to farms in the database. The locations of all farms holding cattle, pigs or sheep are presented in Fig 7. Compiled numbers of the entire population are published online by SJV, but they include no information on the distribution over farms. The data needed for this study was therefore extracted directly from the database and compared as verification against the published data. The data is an estimation of the actual number of livestock in June 2015 [75]. Change in the animal stock of farms is a slow process and the overall differences from 2015 to 2017 data are small (less than 5% both in livestock and animal production, see SJV [76]). There is in total 29 729 Swedish farms holding livestock including cattle, pigs and/or sheep.

Information on the number of cattle and sheep on each farm, are found directly in the extract from the SJV database. For cattle, data is reported either for each individual animal or as number of animals per farm. Information on sheep is directly provided as number of sheep per farm. Regarding pigs, the reported numbers in the database represent the capacity of sow stalls and the number of weaners bred for slaughter. However, some farms only produce weaners for selling to fattening farms. These weaners are not visible in the statistics, which poses an uncertainty. An assumption was made here that all farms that have weaners are included in the list. The total number of pigs on each farm was first set to the number of weaners bred for slaughter plus the number of sow stalls. This number was then scaled with the published number of pigs, according to SJV. Looking at the number of boars, sows, weaning and fattener pigs from the statistics of SJV, a relation between the reported numbers and the actual quantity of animals can be established. Within a few percent, there are 100 sows per boar and there are 8.7 pigs per sow at any time during the year. This procedure meant that weaners were introduced to farms that had registered sow stalls only. Moreover, the scaling implied that the national pig population was maintained in the dataset. The resulting dataset has an uncertainty in the number of pigs at specific farms, but the overall pig population is captured rather well. The discrepancies between the numbers of cattle and sheep extracted from the database and the corresponding published numbers by SJV are less than 10%. The difference is attributed to different methods of handling uncertainties and errors in the database. To harmonize the datasets, the animal populations were scaled according to the published data also for these species. The total animal population was found to be 1 475 525 cattle, 1 356 027 pigs and 594 753 sheep for June 2015. There are uncertainties in these numbers as they are based on an inquiry to farmers and information from previous years.

The numbers of animals at the farms may vary with time over the year due to normal activities as calving, transports and slaughtering. Variations due to the seasonal activities of slaughter, calving and lambing were considered for cattle and sheep. Lambing mainly takes place from January to May while calving is assumed to be evenly distributed over the year. The slaughtering is similarly distributed for the two species; it occurs all year but with increased intensity from August to December. Pigs has a non-seasonal variation over the years and the number is therefore assumed constant over the year. These variations were imposed on the data for June, which resulted in a somewhat varying animal population for the different seasons.

The simulations of airborne disease spread were conducted on each season separately. This allowed for the temporal variations of the livestock to be incorporated by calculating the national mean values for the number of animals of each species for each season. These national values were then used to scale the local animal population on each farm (originally set to the June 2015 numbers) linearly for each simulation depending on season.

### Simulation procedure

The risk for disease transmission from a single farm was simulated by introducing FMD to five randomly selected animals. A simulation was initialized and allowed to continue, using time steps of 6 hours, until the disease on this farm was eliminated, i.e. there were no animals left in latent, subclinical or clinical states. This typically take place when all infected animals reach the states *Recovered* or *Dead*. Note that there might be susceptible animals left on the farm when the disease has been depleted. The number of infected farms were counted and then the same simulation was reiterated. For each season, a total number of 1 000 iterations were conducted to establish results with high precision. This procedure was conducted for each farm in Sweden holding livestock of the three species of interest, implying a total of 119 million simulations. The appendix provides additional details on the model and the rationale behind the choices of the number of iterations and the time steps.

One part of this study involves culling of the infected farm with different delays. This procedure implies that the entire set of livestock at the infected farm was removed; an action that immediately ends the ongoing disease transmission. Culling of a farm therefore constitutes an alternative cause for the infected farm to stop transmitting the disease to neighboring farms. The term *culling delay* refers to the number of days that has passed since the first animal on the farm reached the *clinical* state, which marks the first opportunity for the disease to be discovered at the farm. Another note on the nomenclature, *R* is the *effective reproduction number* and is used to refer to the mean number of infected farms when culling is deployed as a counter-measure.

## Results

For each season, the transmission probabilities between Swedish farms were investigated by means of a large set of simulations for each farm separately. The acquired dataset contains a lot of information and reveals interesting features regarding the risk for an airborne epizootic event in Swedish livestock. The main result of this study is the statistics of the basic reproduction number and the geographical distribution thereof. Table 4 shows basic statistic measures for all seasons and the average for the entire year.

**Table 4:** Statistical measures extracted from the simulation results.

		Winter	Spring	Summer	Autumn	Year
**Sweden**	Mean *R*_*0*_	3.87 (3.68, 4.05)	4.00 (3.81, 4.20)	3.56 (3.39, 3.73)	4.06 (3.86, 4.26)	3.87 (3.78, 3.97)
	Median *R*_*0*_	0.0590 (0.0571, 0.0609)	0.0610 (0.059, 0.062)	0.0540 (0.0523, 0.0557)	0.0660 (0.0638, 0.0682)	0.060 (0.0589, 0.0611)
	Fraction *R*_*0*_ ≤ 0.01	0.204	0.193	0.213	0.187	0.199
	Fraction *R*_*0*_ ≥ 1	0.113	0.113	0.105	0.118	0.112
**Skåne**	Mean *R*_*0*_	11.9 (10.9, 13.0)	12.2 (11.2, 13.2)	11.0 (10.1, 12.0)	12.6 (11.5, 13.7)	11.9 (11.4, 12.5)
	Median *R*_*0*_	0.177 (0.165, 0.189)	0.185 (0.173, 0.197)	0.167 (0.156, 0.178)	0.199 (0.186, 0.212)	0.182 (0.176, 0.188)
	Fraction *R*_*0*_ ≤ 0.01	0.0738	0.0676	0.0792	0.0658	0.0716
	Fraction *R*_*0*_ ≥ 1	0.232	0.235	0.224	0.243	0.234

The resulting values of the basic reproduction number *R*_*0*_ for Sweden presented separately for the four seasons. The corresponding numbers for the subset of farms located in Skåne are presented for comparison. The 95% confidence intervals are presented within parenthesis for the mean and median values.

**Fig 8:**
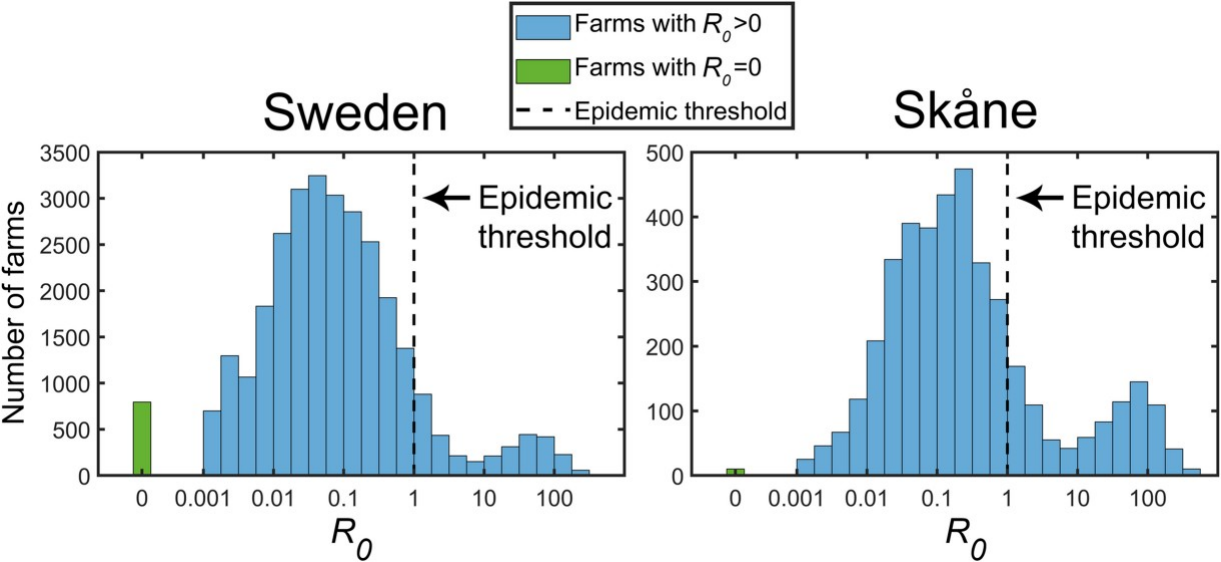
The distributions of *R*_*0*_ values on a logarithmic scale for Sweden and Skåne for winter conditions. The distributions are strongly heterogeneous with the majority of farms well below the epidemic threshold of *R*_*0*_ = 1 and a smaller fraction of farms with *R*_*0*_ in the order of 100. There is a significantly larger risk of epizootics in Skåne than on the national level as the right panel reveals.

Let us first focus on the nationwide results. The yearly averaged *R*_*0*_ for Sweden is 3.87, which means that farms are, when all other farms are uninfected, in average expected to transmit an introduced FMDV to 3.87 other farms. This is, indeed, a relatively high value. In contrast, the median value is only 0.060. The difference between mean and median values is striking. Actually only 11% of the farms have *R*_*0*_ values that exceeds the epidemic threshold of 1.0 while 20% have values less than 0.01. This indicates that there is a small set of farms that acts as strong disease transmitters while the majority of the farms is relatively unlikely to spread the disease at all. A histogram of national *R*_*0*_ values for winter is shown in Fig 8A which reveals a bimodal distribution of *R*_*0*_. The frequency distribution of the number of farms on the *R*_*0*_-axis is actually well described as the sum of two lognormal distributions with a local minimum at *R*_*0*_ = 10. The set of strong disease transmitters is located approximately in the interval 10–200.

**Fig 9:**
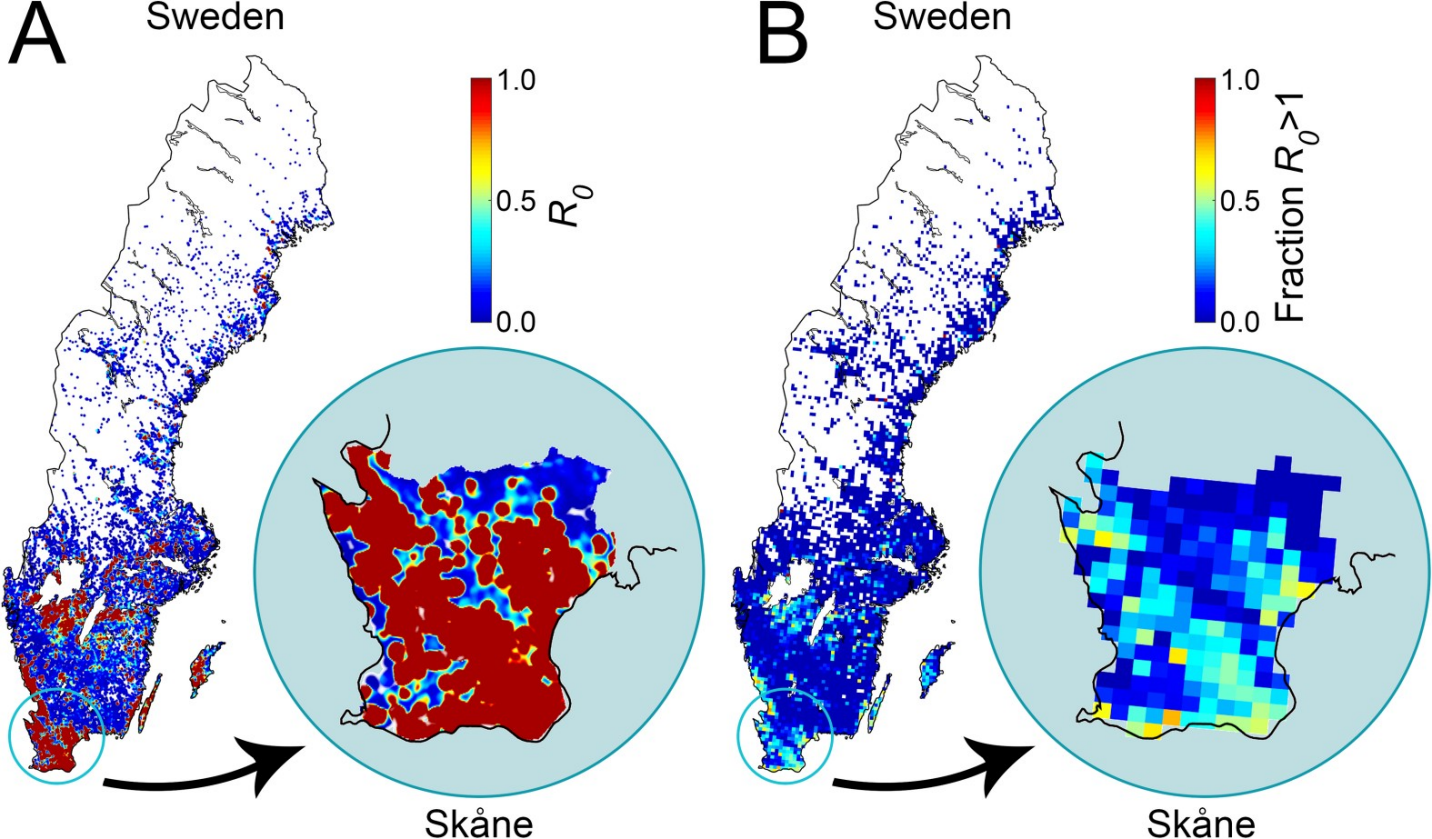
The geographic distribution of the basic reproduction number *R*_*0*_ during winter. Panel A, the values for all of the 29 729 farms have been applied to a high resolved grid using a Gaussian kernel with a standard deviation of 1.25 km to render a geographical representation of the basic reproduction number. Panel B, the fraction of farms with basic reproduction number that exceeds 1 has been counted in each cell of a grid. The borders are provided Natural Earth (public domain).

Another factor of interest is the spatial distribution of the basic reproduction number. A high density of farms increases the risk of disease spread and it is not surprising that high-risk areas are mainly found in southern Sweden. Fig 9 illustrates how the basic reproduction number varies over the country. The data is presented in two different ways. Panel A shows the mean basic reproduction number geographically distributed by applying a Gaussian kernel on each farm with standard deviation of 1.25 km. This method results in a representation of the basic reproduction number that varies smoothly over the country and depends mainly on the closest located farms while farms further away contribute less. Panel B shows the same underlying result using another approach where a grid was placed over Sweden. For each grid cell, the fraction of farms with a basic reproduction number that exceeds one was calculated. The highest risk for disease transmission is predicted in the region Skåne, located in the southernmost Sweden, which has been marked in the figure. Another region with high epizootic risk is located close to Sweden’s two large lakes Vänern and Vättern. Additional smaller regions with *R*_*0*_>1 are also identified as well as scattered hot spots. Northern Sweden has very poor prerequisites to host an FMD epizootic mainly due to the scarce presence of farms. A similar compilation as for the national set of farms was conducted for the subset of farms located in Skåne. The statistics for this region are presented in the lower part of Table 4, in Fig 8B and enlarged as insets in Fig 9 to allow for straightforward comparison with the national data. The frequency distribution and seasonal variation of the basic reproduction number in Skåne follow the same pattern as on the national level, with the exception of a threefold increase in disease spread probability.

**Fig 10:**
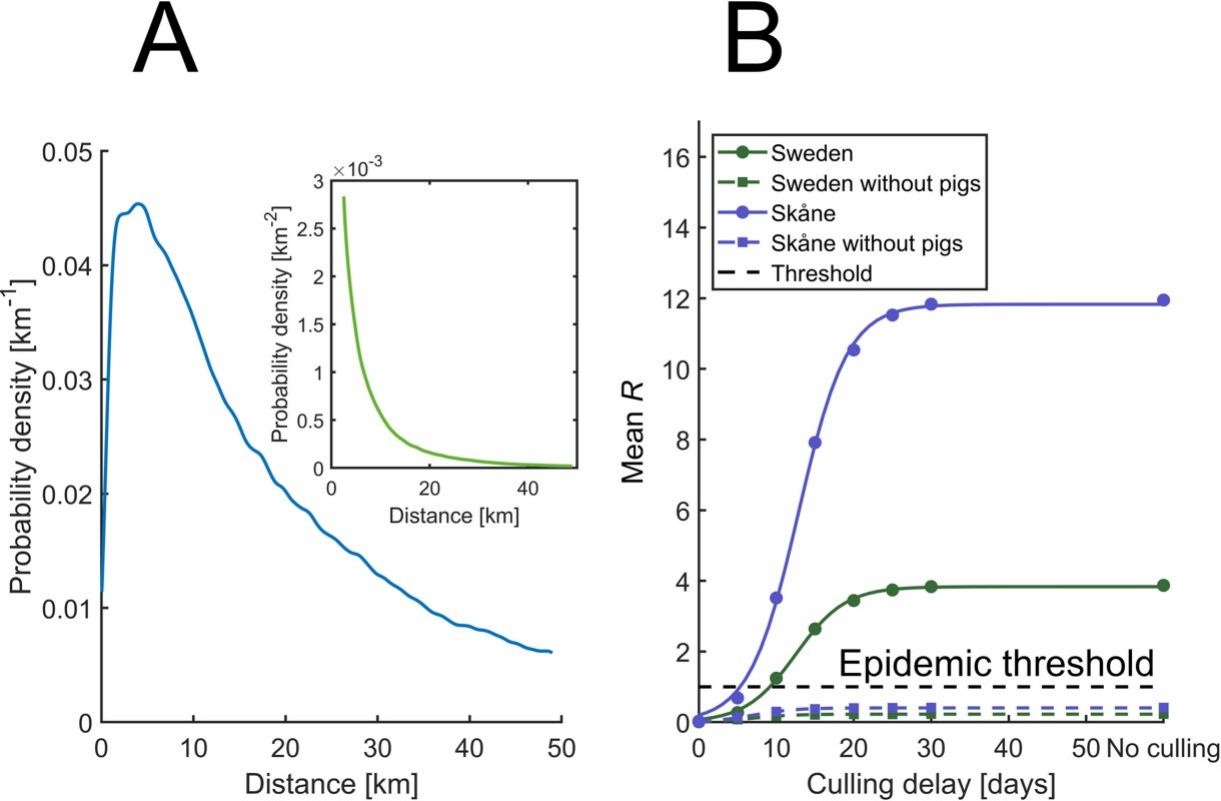
Extracted spatial and temporal distributions of the disease transmission. (A) The probability for disease spread as a function of distance. The low value for distances close to zero reflects the fact that farms are in general rarely located in close proximity to each other. The inset shows the same data with a correction for the increase in area, and therefore the number of farms, with distance. (B) The mean effective reproduction number for all seasons as function of the number of the culling delay. The points form a smooth sigmoidal curve to which logistic functions are fitted and displayed with dashed curves. For each culling time investigated, all farms were used as epizootic seeds during 100 simulations for each season. This implies that each point for Sweden is the mean of 12 million simulations, which results in low numerical uncertainties. The corresponding number for Skåne is 1.6 million. The curves for the set of farms without pigs are depicted with dashed lines. In this case, neither Sweden nor Skåne reached the epidemic threshold.

The simulations were conducted with interfarm networks limited to spread over 50 km distances. This limitation was implemented for efficiency reasons and it is supported by information on historical airborne transmission events. An examination was conducted to confirm that this constraint is indeed valid. All interfarm disease transmissions that took place in the simulations were compiled into a probability density function that depends on the distances. This relation is presented with the blue line in Fig 10A. Now, the number of neighboring farms increases approximately linearly with distance. This fact must be addressed to acquire the dependence for the disease transmission on the distance between two farms. The inset shows the corresponding probability distribution (green) when this factor has been compensated for. This means that the green line in the inset is proportional to the blue line divided by the distance. The inset thereby shows how the probability of disease transmission between two farms decreases with distance while the blue curve is the true indicator on the validity of the 50 km cut-off. The results show that only a minor fraction of the disease transmissions appear to have been discarded. We conclude that the cut-off is motivated and that the impact of the cut-off on the results is negligible.

Fig 10B shows how the effective reproduction number depends on the culling delay (i.e. the number of days after the first clinical sign of FMD on a farm until the livestock is culled). Since it is not possible to establish an analytical solution to the disease transmission system handled here, the behavior of this dependency is also not known a priori. It is found that the curves exhibit sigmoidal shapes that can be well described using a logistic function. The lines in the figure show the logistic functions that where found using an optimization algorithm that minimizes the mean square error. The curves have center values at 12–13 days in culling delay. This implies that the disease spreads most rapidly at this stage of the epizootic.

## Discussion

Animals may be infected on both the intrafarm and the interfarm layer, which is described differently in the model presented here. The intrafarm disease spread is governed by a system of differential equation while the interfarm disease spread uses atmospheric transmission and the exponential infection model. However, it is worth noticing that the somewhat complicated system of differential equation has, indeed, the exponential infection model as analytical solution for a given degree of infection on the farm. This means that the two layers utilize consistent exposure modeling. The exponential infection model has the convenient property of being insensitive to the size of the time steps, which allowed relatively large time steps to be used.

The model presented here incorporates atmospheric dispersion rigorously and in higher detail than previously found in literature in epidemic modeling. Due to the access of a large meteorological data set of high quality, the connection strengths were established in a reliable way under the presented assumptions. Seasonal variations were noticeable but not dramatic; the difference of the mean values of *R*_*0*_ was 15% or lower. Disease transmission is least likely during summer, which is mainly due to meteorological reasons. The high solar heat influx causes a more instable atmosphere, which mitigates the transmission probability, i.e. the connection strength in the nomenclature used here. Note that this finding does not necessarily hold for geographic regions with significantly different climates since a low relative humidity will obstruct the airborne transmission.

It was found that the basic reproductive number varies strongly between the farms, shown in Fig 8. Indeed, a minor subset of the farms are responsible for most of the transmission risk with *R*_*0*_-values from 10 up to 300. Now, strong heterogeneity in disease transmission probabilities have been identified in many other disease transmission systems, with approximately 20% of the individuals causing 80% of the transmissions [77]. For airborne FMD spread in Swedish livestock, the distribution is even more skewed with 5% of the farms causing 91% of the transmissions. Strong transmission farms, with only a few exceptions, are mainly pig farms. There are two main reasons for the high ratio of pigs on the strong transmitting farms. The first is that pigs have substantially higher virus excretion rate than the other species [43, 45, 46]. The second reason is that animals are distributed in different ways in the Swedish livestock. Pigs are concentrated in high numbers on relatively few farms. There are approximately ten times more farms holding cattle than there are farms holding pigs. On the other hand, the average number of pigs, where present, are tenfold higher than the correspond number for cattle. The mean number of pigs on the strong transmission farms is almost 800 while these farms only have a few cattle and sheep in general. The small number of strong emitting farms without pigs hold stocks of sheep and cattle in large number, close to 1 000 in average. The effective reproduction numbers for all farms without pigs were extracted and they are shown in Fig 10B. These farms exhibit small likelihood to, by themselves, harbor an epizootic in any region of the country. The results clearly indicate that the farms holding large pig populations are the main contributors for the risk of an airborne epizootic in Swedish livestock.

Fig 10B shows that the highest risk of disease transmission occurs after 12–13 days. These values are somewhat higher than the values used to establish *β*_*0*_, a result that is actually expected. Several factors contribute to this apparent discrepancy. One strong reason is that the effective reproduction number indicates the probability of transmission to other farms; it is strongly weighted by the excretion rate of the farm populations. Since pigs contribute the most to the spread of the disease from infected farms, their relatively slow intrafarm transmission governs this curve. Further, the strong transmitting farms have in general a large animal population that implies that the maximum source strength of the farm is delayed a few days. The peaks of farms holding no pigs are positioned at lower culling delays (approximately 7 days) but they contribute less to the total amount of interfarm disease spread. The slowest onset of maximum farm infectiousness occurs for farms with a mixture of species. Cattle or sheep may in this case initially be infected by the seeding procedure. Next, the disease is spread to the resilient pigs by the intrafarm route. This process delays the peak infectiousness of the farm by a few days. In contrast, as more farms become infected there are fewer targets available for new infections. This feature causes the maximum likelihood of airborne spread to appear somewhat earlier for strong transmitting farms. Also, a discrepancy in the comparison between these two temporal scales is the latent and subclinical periods since they are not included in the culling delay which time line begins when the first animal enters the clinical state. All these factors contribute in different ways to the relation between the effective reproduction number and the culling delay. The results show that there is only a minor risk for disease spread after three weeks, i.e. farms have passed their peak in infectiousness and already infected the other susceptible farms in its proximity by this time.

The model sensitivity of the included parameters were analyzed and presented in the appendix. It was concluded that the susceptibility and the inhalation rate are the most important parameters. The aerosol size distribution might also have significant impact, but only if the sizes are found to be larger than reported. The susceptibility seems to be the most important parameter to consider since it has a large impact on the results and is not easily determined. The susceptibilities have been found by controlled animal studies, which indeed is a reassuring method. However, the results from these studies are possibly troubled by uncertainties due to the quite small number of animals used in the experimental studies. All epidemiological models are subject to many errors and uncertainties with different origins, and this model constitutes no exception. With this in mind, four case studies were conducted with the aim of identifying possible weaknesses in the modelling system. Admittedly, it is not trivial to draw statistically significant conclusions from such comparisons since the empirical data originates from a stochastic system based on only one realization per case. However, even with that in mind, the case studies provided increased confidence that the model is able to deliver adequate predictions on the airborne disease spread.

The mean value of the basic reproduction number is 12.0 in Skåne and 23.4% of the farms have values that exceeds one. The *R*_*0*_ values are significantly higher than on the national scale. This region has seen outbreaks of FMD in the past. After a series of cases with minor disease spreads, the largest outbreak on Swedish soil took place in 1924–27 [78]. Other relatively large outbreaks occurred 1938–40 and 1951–52 [79]. The last seen outbreak in Sweden was in 1966 which is interesting since it is suspected to have originated from a laboratory in Denmark and was introduced to Skåne by means of airborne transmission over Öresund that divides Denmark and Sweden [79, 80]. The close relations and proximity to Denmark, also with a high density of farms, implies an increased risk of introducing FMD. If present, FMD is likely to spread quickly in this region.

As concluded, the simulations resulted in relatively high mean *R*_*0*_ values driven by the set of strong disease transmitting farms. It should be noted that these values are based on disease spread with no interventions whatsoever (except for the minor study using culling with different delays). The disease was allowed to spread freely until there was no active infection present anymore. In case of an actual outbreak, most countries will be able to act quickly to disrupt the disease transmission in an early stage. The results presented here underlines the importance of not only isolating infected farms but also to cull the animals since putting the farm into a quarantine state will not affect the airborne transmission. The risk of disease transmission by the airborne route between farms is highest approximately 5–20 days after the disease becomes detectable at the farm. Taking into account that there is a delay until a disease is detected, this leaves a short time span to conduct a culling operation.

This study used parameter values that are relevant to serotype O which has been dominating outbreaks on the European scene. It should be noted that the dynamic properties involved in airborne spread of FMD might differ significantly between different serotypes. For instance, the excretion rate was found to vary with up to two orders of magnitude depending on the strains [81].

The basic reproduction number is a measure of the likelihood of disease spread within the population. Indeed, in a well-mixed homogeneous population there is an epidemic threshold at *R*_*0*_ = 1. The basic reproduction number serves as an indicator of the systems inclination to host an epidemic even in a heterogenic network. However, to quantify the expected magnitudes of outbreaks for such systems it is necessary to weigh the basic reproduction number of each farm by their susceptibilities of becoming infected. Such a study would be a suitable continuance to the results presented here. Besides producing a quantitative and spatially distributed risk map of airborne transmission of FMD, this study also resulted in a weighted and directed transmission network that may serve as an excellent starting point for that purpose.

## Supporting information

S1 Appendix Model properties. Additional information regarding the precision and accuracy, parameter sensitivity and case studies.
(DOCX)

S1 Data (XLSX)

S1 Fig (TIF)

S2 Fig (TIF)

S3 Fig (TIF)

S4 Fig (TIF)

S5 Fig (TIF)
