# Correction: Multilevel model for airborne transmission of foot-and-mouth disease applied to Swedish livestock

**DOI:** 10.1371/journal.pone.0244374

**Published:** 2020-12-16

**Authors:** Oscar Björnham, Robert Sigg, Jan Burman

In the Intrafarm disease spread subsection of the Methods, there is a formatting error in the equation in the second paragraph. Please view the complete, correct equation here:

$$\begin{array}{l} \dot{\bar{S}}=-\frac{\beta_{0}}{N}(\bar{\rho }\bar{\Lambda_{I}})\circ \bar{\Lambda_{S}}\circ \frac{\bar{\Lambda_{F}}}{\Lambda_{F,C}}\\ \dot{\bar{E}}=\frac{\beta_{0}}{N}(\bar{\rho }\bar{\Lambda_{I}})\circ \bar{\Lambda_{S}}\circ \frac{\bar{\Lambda_{F}}}{\Lambda_{F,C}}-\bar{\varepsilon }\circ \bar{E}\\ \dot{\bar{I}}=\bar{\varepsilon }\circ \bar{E}-(\bar{\gamma }+\bar{\chi })\circ \bar{I}\\ \dot{\bar{J}}=\bar{\gamma }\circ \bar{I}-(\bar{\delta }+\bar{\eta })\circ \bar{J}\\ \dot{\bar{R}}=\bar{\chi }\circ \bar{I}+\bar{\eta }\circ \bar{J}\\ \dot{\bar{D}}=\bar{\delta }\circ \bar{J} \end{array}$$
