# Supplementary material for: Multilevel model for airborne transmission of foot-and-mouth disease applied to Swedish livestock
Source: PLoS One. 2020 May 26;15(5):e0232489. doi: 10.1371/journal.pone.0232489 (PMC7250458; doi:10.1371/journal.pone.0232489)
Supplement: S1 Appendix — Additional information regarding the precision and accuracy, parameter sensitivity and case studies. (DOCX) [file pone.0232489.s001.docx]

# Model properties

A comprehensive model and associated methods have been assembled to be able to estimate the probability for an FMD epizootic. As a compliment to the presented model features, three technical perspectives are collected in this appendix to provide a deeper and more detailed understanding of the model. First, since the model utilizes a stochastic scheme, the model outcome is expected to be sensitive to the number of iterations used. Moreover, the choice of time step in the simulations could have impact on the results. A numeric study was conducted on this issue, which is described below. Second, no controlled empirical data set exists that can be used for validation purpose with statistical significance on an epizootic level. Post-analyses of live outbreaks shed light on the issue, but are always troubled by a lack of important information that cannot easily be retrieved in the aftermath. Even so, these analyses do provide insight in the development of FMD disease spread. Four different airborne outbreaks from the UK 2001 epizootic have been used as case studies here. The pre-requisites were reproduced and the real-case outcomes are compared with the predictions made by simulations. Finally, a sensitivity study is presented. The model sensitivity, with regard to 13 different model parameters, was quantified to recognize the relative importance of the parameters.

## Accuracy and precision

A standard measure of the risk for an epidemic or epizootic outbreak is the basic reproduction number *R_0_*. The basic reproduction number is the expected number of individuals, in an otherwise healthy population, to which one infected individual will transmit the disease. The individuals that were infected by the first individual will, in turn, be able to transmit the disease further on. Note that any such secondary disease transmissions are not included in the calculation of *R_0_*. The value of *R_0_* is of great interest since it indicates if an outbreak is probable to occur or not. If *R_0_* is below 1, the disease may still be transmitted but it will most likely be extinguished rapidly. If *R_0_* instead is above 1 there is a high risk that the disease will spread widely in the population.

In the case discussed here, the population is constituted by the farms and each farm can be attributed a specific *R_0_*‑value. These values will differ between neighboring farms due to dissimilar animal populations and the likelihood of local wind directions, i.e. wind roses, in combination with the positions of the farms in a heterogenic farm distribution. As a general trend over larger areas, *R_0_* may change as a result of both meteorological conditions and density of farms. In case a deterministic disease transmission model would have been used, the *R_0_*‑value of a farm could be calculated by initiating the disease on the farm, running the simulation and counting how many farms that were infected directly from the farm under investigation. However, the epizootic model described here utilizes a Monte Carlo approach, i.e. stochastic modeling. This implies that an *R_0_*‑value from one simulation is a stochastic sample from a probability distribution. To obtain statistically significant values, *R_0_* is calculated as the ensemble mean for a number of simulations, here referred to as *iterations*. The central limit theorem states that *R_0_* calculated this way is a normally distributed stochastic sample. Further, the variance of *R_0_* will scale with the reciprocal of the number of iterations. That is, a larger number of iterations will imply less uncertainty of the *R_0_* samples. The number of iterations needed to acquire a certain precision is unknown a priori. The transmission model is based on a system of differential equations and discrete time steps were used to find the numerical solution. The temporal step size is another factor to consider in a simulation of a time evolving process. Smaller time steps imply better results in general. In fact, when the time step increases it may introduce a numerical bias into the results. Both the number of iterations and the temporal step size will influence the results. The best results are obtained using short time steps and a large number of iterations. However, small temporal steps and a large number of iterations can be computational expansive which means that the dependence on these two factors for the precision and accuracy of *R_0_* is of great interest.

To summarize, we have two factors that control how the simulation procedure is conducted that affect the *R_0_* values. The number of iterations will determine the variance, i.e. the precision, while the temporal step size will determine the bias in the expectation value of *R_0_*, i.e. the accuracy. A numerical investigation was conducted to find the sample size required to obtain estimates of *R_0_* with an acceptable uncertainty and to illuminate the bias caused by the temporal step size.

A specific farm was randomly selected to constitute the source of infection and this farm was used consistently in the entire investigation. The estimated mean value of *R_0_* was calculated using one million iterations distributed between three temporal step sizes of 1, 12 and 24 hours. Longer time steps than 24 hours have not been included in this test since the FMD disease model have transition periods close to this time length and thereby renders such time steps unsuitable.

Panel A in Fig S1 shows that the size of the time steps has little influence on the results even though a weak tendency to a positive bias is noticeable as the time step increases. The number of iterations does not influence the expectation value of *R_0_* but it strongly affects the variance of the results as is seen in both panels. The right panel illustrates how the variance of the *R_0_*‑value changes with the number of iterations. The solid curves show the interval of one standard deviation in both directions from the expectation value of *R_0_* which is depicted with the dashed line. If one specific farm is of interest, it can be argued that the uncertainty resulting from 100 or fewer iterations is too substantial for robust conclusions to be drawn. In this case it is recommended to use at least 1 000 iterations. However, the prime result of this paper is to investigate how the reproduction number behaves in the ensemble of Swedish farms. Variations over geographical regions will be studied and not individual farms. The results from many farms will be congregated which implies that their common properties are based on the total amount of iterations for the conglomerate. The estimations of *R_0_* over geographical areas will therefore be robust with considerable fewer iterations per farm.

Fig S1. The precision and accuracy of estimations of the basic reproduction number, R_0_, depending on the time step and number of iterations used. (A) The estimate of the R_0_ values (black dashed line) calculated as the mean of more than one million iterations distributed between the three time step values 1, 12 and 24 hours. There is a slight increase of R_0_ with increasing time steps due to numerical effects. However, this effect is below 2 % when the time step increases from 1 to 24 hours. Moreover, the panel shows the interval of the mean plus/minus 1 standard deviation for different sample sizes, i.e. the number of iterations. (B) The variance decrease with the number of iterations. The markers show simulated values from the same samples as the left panel while the lines are the theoretic prediction, i.e. variance scales linearly with the number of iterations, using the variance at 50 iterations as anchoring points. The values with 100 and 1 000 iterations agree well with this prediction.

The conclusion from the study of the accuracy and precision in the estimation of the basic reproduction number is therefore that at least 1 000 iterations should be conducted for a single farm while for a mean value of many farms it might be sufficient with ~100 iterations. Even so, to ensure high precision in this study, the transmission risk was calculated using 1 000 iterations for each farm. The time step may arguably be set to 24 hours when no culling occurs. However, the temporal aspect becomes important when culling is present. Therefore, a time step of 6 hours was consistently used in this study.

## Real case comparisons

Since no empirical data set based on a large-scale controlled epizootic can be staged in practice, it is difficult to conduct a validation with significant statistical measures for the entire system. Now, the model is constituted by several components that are populated by many parameters, and the reliability of the model as a whole is based on the reliability of those components and the parameters. The system as such is novel, but the components, parameters and methods are in many ways established, well tested and widely used in the literature. And even though a validation with significant statistical measures cannot be conducted for the entire system, it is illuminating to apply the model to documented real cases and compare the outcomes. To do so, the model described here was compared against four different outbreaks during the 2001 UK epizootic. In all cases, the distribution of farms was replicated and the reported meteorology during the event was handled by creating wind roses that correspond to the relevant period of time. To obtain statistically significant results for the model, the case was simulated using 10 000 iterations.

Gloster et al. present, in excellent detail, three case studies of airborne spread of FMD during the 2001 UK epizootic ([Gloster et al., 2005](#_ENREF_2)). We have replicated all these cases individually and compared model predictions to the reported outcome. The first case targets the spread in the Longtown area that originated from the Smalmstown Farm and began in late February. At March 2 over 300 cows were clinically infected and analyses show that 9 of 145 other local farms were likely to have been infected by airborne transmission from Smalmstown Farm. The second case is about the Hall Farm in the Penrith area at the turn of the month between May and June. Hall Farm held only 24 cows of which 20 were found to be infected. Although constituting a relatively weak source term the disease seems to have infected 3 of 24 neighboring farms. The third case addresses the Marshall Green Abattoir in the Witton-le-Wear region in late February. Three cattle and 31 pigs were post-mortally diagnosed with FMD and could have been contagious for 3-5 days. 8 of 72 farms located in close proximity are suspected to have been infected by airborne transmission from the abattoir. As a fourth case study, the Burnside case was investigated. The pig farm held 527 pigs, and it was infected late January or early February. When samples were taken on February 24, 90 % of the animals tested positive for FMD antibodies. The strong source term caused possible airborne spread to a number of neighboring farms. Twelve neighboring farms were soon reported to be infected. Of these twelve, two could be dismissed as receivers of airborne transmission from the Burnside Farm, and there are inconclusive information for three additional farms. The case has previously been analyzed in detail ([Alexandersen et al., 2003](#_ENREF_1); [Gloster et al., 2003](#_ENREF_3); [Mikkelsen et al., 2003](#_ENREF_4)) from where outbreak information and prerequisites, i.e. farm data and meteorology, for the event are obtained. In lieu of detailed information regarding unaffected farms, the case was set up using 60 additional farms evenly distributed in nearby regions indicated to hold animal farms. Since little information was provided regarding the animal population on the farms, it was set to 100 cows and 100 sheep at each farm except for Burnside with its 527 pigs and Prestwick Hall Farm with 100 cows, 30 pigs and 320 sheep.

The median numbers of infected farms, and the percentage of simulations that gave rise to disease spread, were identified for each set of simulations and are collected in Table S1. The simulation results indicate that disease spread to the neighboring farms was indeed possible in all four cases. There was a good agreement also in the magnitude of the outbreaks in three of the cases. The largest discrepancy was found in the Penrith case where the model predicted that disease spread was possible but that it was unlikely. This is also the case with the fewest documented transmissions.

Table S1. Results from the case study.

|  | **Infected farms** | |
| --- | --- | --- |
| **Case** | **Model** | **Reported** |
| Longtown | 2.3 (92 %) | 9 |
| Penrith | 0.12 (11 %) | 3 |
| Witton-le-Wear | 3.8 (96 %) | 8 |
| Burnside | 12 (100 %) | 7-10 |

The case study showed satisfactory agreement between model outcome and reported disease spread. The model outcome is presented as mean number of infected farms with the percentage of simulation giving rise to disease spread in parenthesis. The statistics is based on 10 000 simulations for each case study.

Now, it is not straightforward to interpret the results from this kind of comparison. As already mentioned, a large number of simulations were conducted to obtain a reproducible distribution of the predicted disease spread. However, there is little reason to expect that the actual outcome of an epizootic represent the expectation value of such a distribution. It is one realization of a stochastic system presumably with strong variations. Similar prerequisites may very well have resulted in no disease spread, and such cases are unfortunately not expected to be highlighted in analyses. This would imply that specific case studies might be troubled by a positive bias, i.e. that they show greater disease spread than what was really expected. Therefore, it is difficult to set up quantitative requirements for a statistically robust validation process. It is, however, reasonable to require that the model outcome indicate that a disease spread was indeed likely to occur without greatly over-predicting the reported spread. As mentioned, one should be careful not to exaggerate the interpretation of this kind of comparison. Even so, it is clear that the outcome found here does not indicate that that there are any significant discrepancies and the case comparison strengthens our confidence in the model.

## Sensitivity analysis

The model described and used in this study includes several processes and many parameters. Even though their purposes are distinct and clear, it is not trivial to understand their separate contributions and significances to the model outcome. To shred light upon this question and provide an improved understanding of the model properties, a sensitivity study was conducted that is described in this section.

First, the model outcome was calculated for the so-called *base line* case, which means that no parameters were changed. To scrutinize the model sensitivity of the included parameters at the intrafarm layer, the mean number of infected farms was recalculated with systematic variations in the parameter setup. A farm in southern Sweden (PPN 178) was selected to constitute the source for this exercise. It has 1 329 neighboring farms within its reach of 50 km. The farm was populated with 60 animals evenly distributed between the species yielding a basic reproduction number of 2.9. All relevant parameters presented in the intrafarm disease spread were individually varied on a logarithmic scale by steps of two. The base line value for one parameter was multiplied by a factor of 1/8 and the mean number of infected farms was calculated. This process was then repeated for factors of 1/4, 1/2, 2, 4 and finally 8. The parameter value was then reset to the base line value and the procedure was repeated for all other parameters separately, i.e. it is a one-at-a-time sensitivity study.

The model sensitivities of the parameters involved in the atmospheric transport between farms, i.e. the interfarm layer, were quantified with a similar but slightly modified technique. The impact of these parameters on the exposures at other farms was first calculated, using the same factors as for the intrafarm layer. These results were thereafter readily translated into relative changes in the mean number of infected farms. A note on the nomenclature, the term *exposure* is used for the time-integrated pathogen concentration (normalized with the amount of released pathogens) to improve the readability. This entity can also be regarded as the time-integrated probability density for the atmospheric transport and has the unit of time/volume.

In conclusion, all parameters were varied similarly and the same output (relative change in the mean number of infected farms) was collected for them. This allowed the model sensitivity for all parameters to be directly compared against each other.

### Intrafarm layer

The model sensitivity on the intrafarm level was analyzed by varying eleven relevant parameters. 10 000 simulations were conducted for each parameter variation to obtain the corresponding mean number infected farms with high statistical precision. These numbers were then divided with the results from the base line case whereby the relative impact for each single parameter was found. Fig S2 shows the outcome, with no culling in the left panel and with culling after 10 days in the right panel.

Fig S2. The model sensitivity of the parameters on the intrafarm layer for an infected farm.

The parameters $\Lambda_{F}$ and *s* have the highest impacts on the mean number of infected farms. These two parameters are coupled to the risk of becoming infected when exposed, and they are actually present on both the intrafarm and interfarm layers. They are multiplied in the infection model used on the interfarm layer wherefore variations in their values have equal impact on the probability for an exposed neighboring farm to become infected. They are also multiplied on the intrafarm layer. However, the Λ*_F,C_* value is used in the denominator for the rate equations on the intrafarm layer to obtain a dimensionless scaling parameter *β_0_*, eq. (4). This model feature means that the variations in Λ*_F_* have no explicit effect on the intrafarm disease spread. Actually, the value of the scaling parameter depends on the other parameters which means that altering Λ*_F_* is equivalent to altering the fitting of the *β_0_*-parameter on the intrafarm level due to the normalization process. The difference between the parameters on the intrafarm layer has negligible effect when the probability that the all animals on the farm becomes infected is high. When this is the case, only their contributions on the interfarm layer that matters. On the other hand, for low factors for s, the intrafarm disease spread becomes limited and the disease spread drops of significantly. It is found that the base line case has an animal population that in general becomes completely infected. There is not much marginal though, and as s is lowered more and more animals remains uninfected. This is the reason why the sensitivities for s and Λ*_F_* diverge for factors lower than 1. The model also shows the strongest sensitivity for the parameter *s* when culling is present. Since the culling occurs before all animals on the farm have had time to become infected, the parameter *s* has a strong influence on the disease spread and becomes more important than Λ*_F_* for all factors.

Increases in the source term parameters *i* and *j* have relatively high impact on the model outcome, while the model is less sensitive to decreases in their values. The parameter $\chi$ describes how large fraction of the animals that recovers without entering the clinical state. The mean number of infected farms declines significantly for an increased value of this parameter while a decrease has only minor influence. The parameter *η* determines, to a high degree, how quickly animals leave the clinical state. A decrease in *η* implies that infectious animals remain in that state a longer time and thereby increase the risk of disease transmission. This parameter imposes significance to the results especially for low values when no culling is present. In the case with culling after 10 days, many animals have not yet likely to have exited the clinical state and they are therefore not affected by this parameter. The remaining parameters all influence the model outcome but more moderately.

### Interfarm layer

FMD spreads between farms by means of atmospheric dispersion on the interfarm layer. The purpose of the atmospheric transport simulations is to, given an infected farm, estimate the exposure at neighboring farms. The atmospheric dispersion model used here has been validated with a number of experimental field trials. Even though the dispersion model relies on the modeling of many physical processes, there are only two parameters specifically coupled to this case and thereby relevant for a sensitivity analysis: the aerosol size distribution and the decay rate of the virus. To quantify the impact of these two parameters, the exposure field were recalculated with variations in their values using dispersion simulations with the same meteorology as used for the exposure field shown in Fig 5, i.e. stability class E and a wind speed of 2 ms^-1^.

The size of an aerosol determines the settling velocity and thereby how fast the droplet deposit on the ground, which disables it from spreading the disease. The aerosols in the simulations have random sizes (diameters) drawn from a lognormal distribution, which is defined by a mean and a geometric standard deviation. The latter is kept unchanged in this sensitivity study while the mean is varied with the same set of factors as for the intrafarm layer. That is, the impact of the aerosol size distribution was investigated by running the atmospheric dispersion model with six different values of the mean aerosol size to obtain the corresponding exposure fields, which were compare with the results for the base line case. The variations in the mean value of the aerosol size gave rise to the distributions presented in Table S2.

Table S2. Aerosol sizes.

| **Relative aerosol sizes** | **d < 3 μm** | **3 μm < d < 6 μm** | **d > 6 μm** |
| --- | --- | --- | --- |
| 1/8 | 99 % | 1.4 % | 0.1 % |
| 1/4 | 90 % | 8.0 % | 1.5 % |
| 1/2 | 67 % | 23 % | 9.5 % |
| 1 (Base line) | 34 % | 34 % | 33 % |
| 2 | 9.9 % | 24 % | 66 % |
| 4 | 1.6 % | 8.3 % | 90 % |
| 8 | 0.1 % | 1.4 % | 98 % |

Seven different size distributions were used for sensitivity test. The distributions were altered by changing the mean of the lognormal distribution, d, with a factor of two for each step. The fraction of the aerosols within three intervals (the same intervals as was used in the referred literature for the base line settings discussed in the section “Atmospheric Dispersion Modeling”) is presented.

An exposure field, for the region up to the distance of 50 km, was compiled by means of atmospheric dispersion modeling for each aerosol size distribution. The exposure was averaged over all directions to establish a mean dependence on the distance. These results are presented in the top left panel in Fig S3. By normalizing the exposures against the base line case, we obtained the relative change of the exposure, shown in the top right panel. The largest impact is found in proximity to the source and is most pronounced in the cases with large aerosols. The relative change in exposure is almost constant after a few km.

A similar study was conducted where the virus decay rate was changed instead. The dependence of the mean exposure with distance is shown in the bottom panels in Fig S3. The impact of the decay rate increases strongly with the distance since the travel time becomes longer. However, the exposure is very small at great distances and the probability of disease transmission is therefore small. A large change in the relatively exposure at long distances therefore only has a small effect on the total disease transmission. Actually, the two model features display diametrically different dependences on the distance. Aerosol sizes induce an immediate effect close to the source while the decay rate has an effect that is negligible close to the source but increase close to exponentially with distance.

Fig S3. The exposure dependence on distance for variations of aerosol size distributions (the two top panels) and for variations of the decay rate (bottom two panels). The impact of the aerosol sizes is mainly manifested in the region close to the source that show a rapid decrease in the exposure field with increased aerosol sizes. The decay rate causes an exponential decrease in the survival probability for each aerosol with time. In combination with the dynamic development of the atmospheric transport, this feature gives rise to almost straight lines in the bottom right panel.

For any given farm position, the received dose can be determined by the local exposure, the amount of released pathogens and the present livestock at the farm. Since the dose is directly linked to the probability of disease transmission, the relative change in probability to infect the farm can be determined from the exposure fields.

The goal here is to establish the mean number of infected farms in the entire region, which equals the sum of the probabilities for infection for each farm. Now, the probability for infection are in general very low. The farms in Sweden have in average 1 200 neighboring farms they can potentially infect. The mean value for the basic reproduction number in Sweden was found to be ~4, which means that the each farm has a mean probability of about 1/300 to become infected. Now, for low infection probabilities, the exponential model for an animal becomes effectively linear,

$P_{\inf}\left(n\right)=1-e^{-ns}\approx ns$. (S1)

where *n* is the dose, i.e. number of pathogens and *s* is the susceptibility of that animal. The dose equals the exposure, *e*, at the farm multiplied with the number of released pathogens, *κ*, and the inhalation rate of the animal, Λ.

$n=e\kappa\Lambda$ (S2)

Now, the probability for any animal at the farm *b* to become infected is

$P_{\inf,farm}\left(n_{b}\right)=1-\prod_{k} \left(1-P_{\inf}\left(n_{b}\right)\right)\approx\sum_{k} P_{\inf}\left(e_{b}\kappa\Lambda_{k}\right)=e_{b}\kappa\sum_{k} \Lambda_{k}s_{k}=e_{b}\kappa\sigma_{b}$ (S3)

where the index *k* loops over all animals at the farm and $\sigma_{b}$ is a measure of the susceptibility of the farm as a unity and is independent of the exposure. The average number of infected farms in total, *P*, becomes

$P=E\left(\sum_{b} P_{\inf,farm}\left(n_{b}\right)\right)=E\left(\sum_{b} e_{b}\kappa\sigma_{b}\right)$ (S4)

where *E* is the expectation value and the index *b* loops over all neighboring farms. There is no correlation between the exposure and the susceptibility of a farm, which means that this expression can be rewritten as

$P=E\left(\sum_{b} e_{b}\kappa\sigma_{b}\right)=\kappa\bar{\sigma}\sum_{b} e_{b}$ (S5)

The relative change in the average number of infected farms when the exposure field is altered compared to the base line case becomes a function of the exposures only,

$\frac{P_{Altered}}{P_{Baseline}}=\frac{\sum_{b} e_{b,Altered}}{\sum_{b} e_{b,Baseline}}$. (S6)

The sum of all farm exposures is therefore a direct measure of the relative change in expected infected farms. Now, the expected number of neighboring farms depends on the distance. Therefore, the requested measure, i.e. the relative change in mean number of infected farms, depends on the exposure fields but also on the probability for neighboring farms at different distances. To account for this, the distribution of distances between all Swedish farms was collected. It is displayed in Fig S4 for distances up to the cut-off limit of 50 km.

Fig S4. All distances to neighboring farms for all farms in Sweden were compiled into a histogram, with bin width of 1 km, and normalized against the number of farms. The distribution is smoothly increasing with distance and it is depicted in orange in the figure. Since the area for each bin increases linearly with the distance, the average number of farms is expected to increase with distance. The relation between the farm density and the distance is found by dividing the number of farms in each bin with the corresponding area. This entity, depicted in green, is actually decreasing with distance presumably because of geographic and anthropological reasons. For instance, the farming prerequisites are not isotropically distributed; there tends to be many farms in proximity to each other at regions with good conditions.

The exposure fields were discretized similarly as the farm distances. That is, the region was divided into 50 different distances, i.e. circular bands with widths of 1 km. The relative change in total farm exposure is found by summing the exposures multiplied with the number of farms at each distance bin, and normalize this against the corresponding total farm exposure for the base line case, i.e. eq. (S6). The results are presented in Fig S5 for both parameters.

Figure S5. The model sensitivity of the atmospheric transport parameters. The interfarm infection probability decrease rapidly with increasing aerosol diameter d.

### Conclusion

As a general observation, the mean number of infected farms changes slower than the parameter values for both the intrafarm and interfarm layers. For most parameters, this difference is quite substantial. The are two exceptions: the susceptibility and the aerosol diameter. The transmission probability increase strongly with the susceptibility if the spread on the intrafarm layer is limited so that not all animals are expected to become infected. This is mainly the case for low susceptibilities or when culling is present. The identical impacts of the susceptibility and the inhalation rate on the interfarm layer are linear for low doses but decreases as the infection probability becomes substantial. The discrepancy in the sensitivities between these parameters is caused by their different impacts on the intrafarm layer only.

It is worth noting that the diameter of the aerosols is altered in this sensitivity study. Aerosols have setting velocities, i.e. gravitational pull downward, that scales with its mass. Since the mass increases with the diameter raised to the 3th power, an increase of the diameter by 8 implies an increase in the aerosols mass by a striking factor of 512.

Note that the translation between exposure fields and relative change in the mean number of infected farms relied on a simplification of the infection model. It is clear that this simplification is justified and valid in the vast majority of cases. However, this method can induce an overestimation of this metric for neighboring farms in the closest proximity to the source. When the infection probability increases, the derivative thereof decreases and approaches zero as the probability approaches one. This effect is illustrated by the curve for $\Lambda_{F}$ on the intrafarm layer where this simplification has not been used. The curve shows a linear behavior for low values, which is in line with the simplification in eq. (S1). As the inhalation rate increases, the infection probability also increases and the slope for the mean number of infected farms decreases somewhat (on a linear scale). The model sensitivities of the two parameters on the interfarm layer therefore technically constitute limiting values.

In conclusion, it is found that changes in most of the model parameters will induce moderate effects on the model outcome, i.e. the partial derivatives for the parameters are lower than one. The two exceptions to this conclusion is i) the susceptibility, which may impact the disease spread on both layers and ii) the aerosol diameter, which, if increased, will result in a rapid decline in the disease transmission.

# References

Alexandersen, S., Kitching, R.P., Mansley, L.M., Donaldson, A.I., 2003. Clinical and laboratory investigations of five outbreaks of foot-and-mouth disease during the 2001 epidemic in the United Kingdom. Vet Rec 152, 489-496.

Gloster, J., Champion, H., Mansley, L., Romero, P., Brough, T., Ramirez, A., 2005. The 2001 epidemic of foot-and-mouth disease in the United Kingdom: epidemiological and meteorological case studies. The Veterinary Record 156, 793-803.

Gloster, J., Champion, H., Sørensen, J., Mikkelsen, T., Ryall, D., Astrup, P., Alexandersen, S., Donaldson, A., 2003. Airborne transmission of foot-and-mouth disease virus from Burnside Farm, Heddon-on-the-Wall, Northumberland, during the 2001 epidemic in the United Kingdom. The Veterinary Record 152, 525-533.

Mikkelsen, T., Alexandersen, S., Astrup, P., Champion, H., Donaldson, A., Dunkerley, F., Gloster, J., Sørensen, J., Thykier-Nielsen, S., 2003. Investigation of airborne foot-and-mouth disease virus transmission during low-wind conditions in the early phase of the UK 2001 epidemic. Atmos Chem Phys 3, 2101-2110.
