# Supplementary figures and images for: Multilevel model for airborne transmission of foot-and-mouth disease applied to Swedish livestock
Source: PLoS One. 2020 May 26;15(5):e0232489. doi: 10.1371/journal.pone.0232489 (PMC7250458; doi:10.1371/journal.pone.0232489)

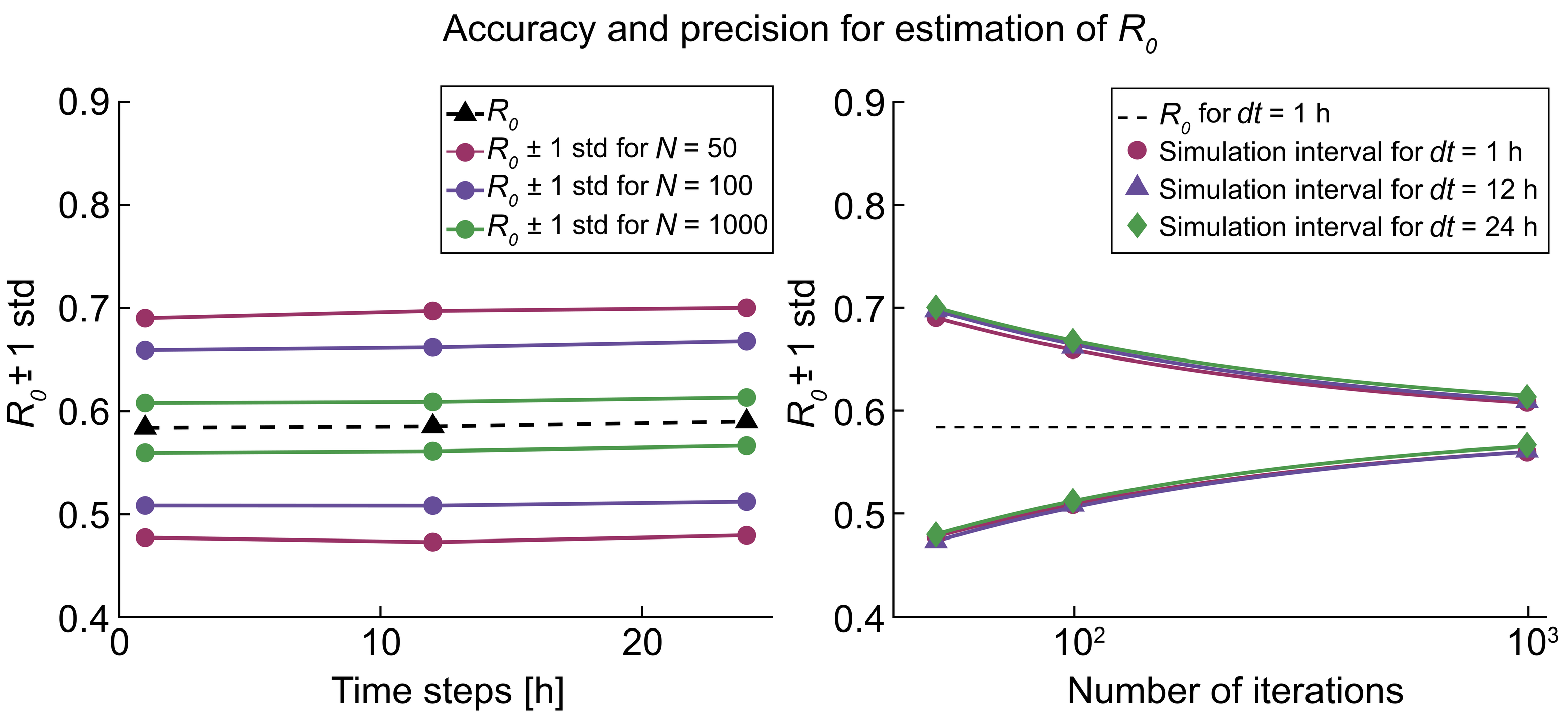

Supplement: S1 Fig — (TIF) [file pone.0232489.s003.tif]

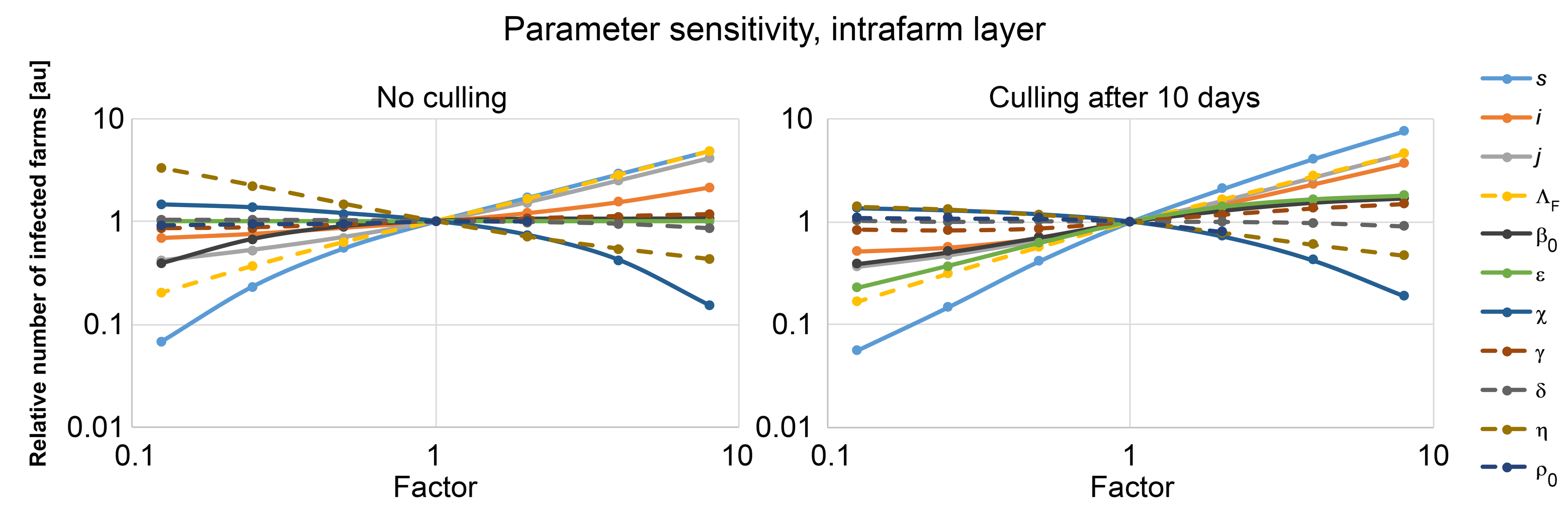

Supplement: S2 Fig — (TIF) [file pone.0232489.s004.tif]

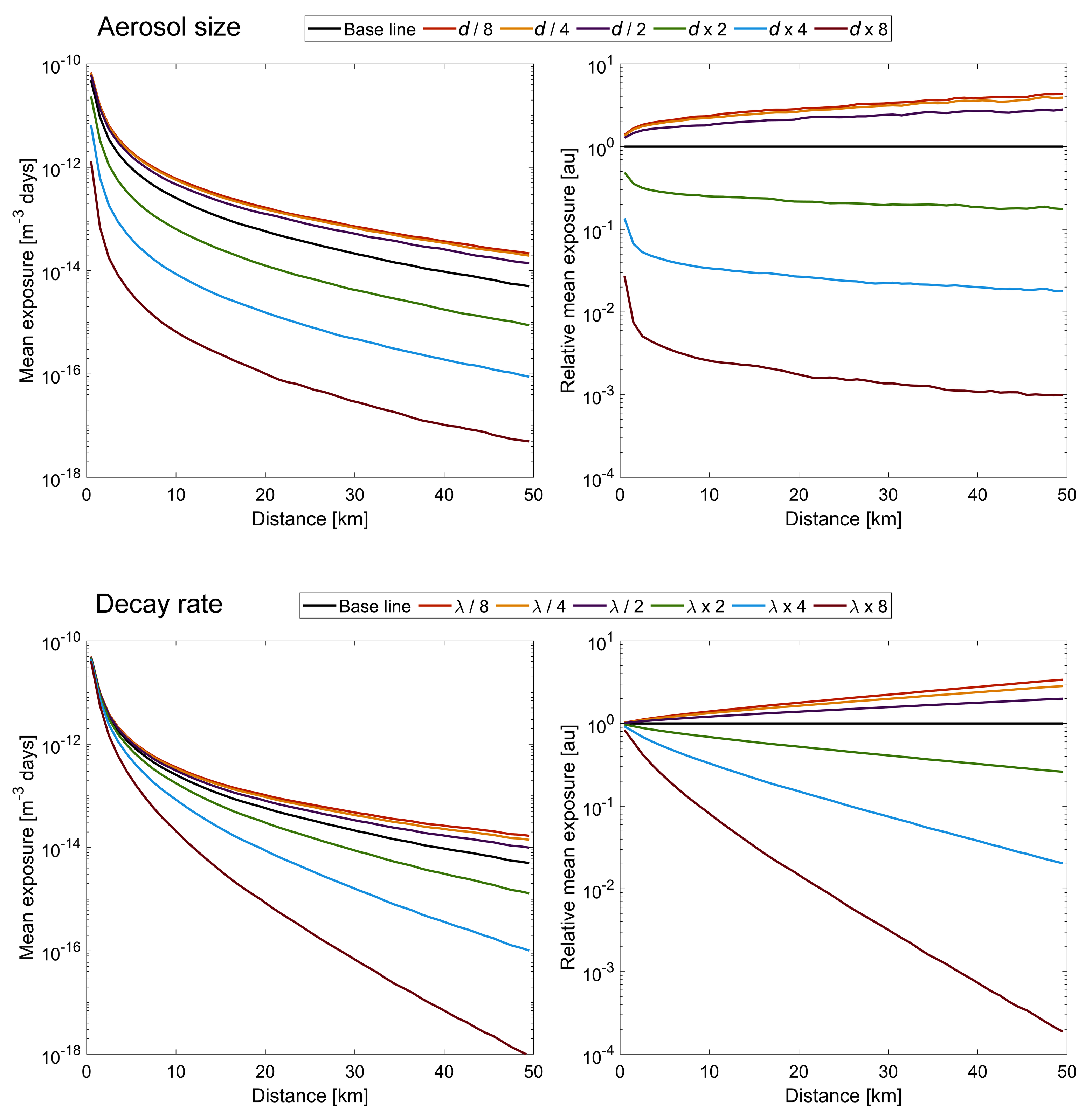

Supplement: S3 Fig — (TIF) [file pone.0232489.s005.tif]

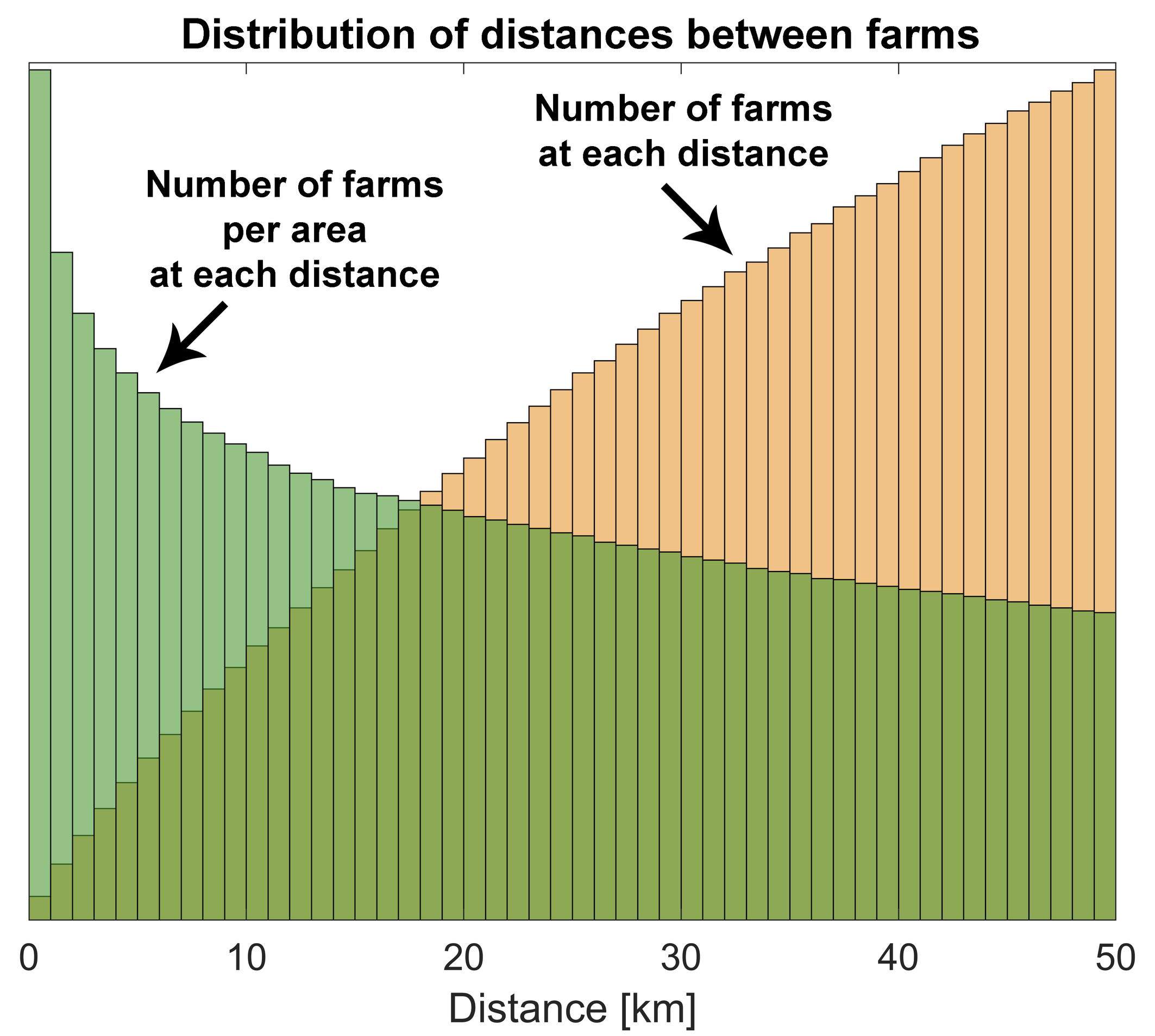

Supplement: S4 Fig — (TIF) [file pone.0232489.s006.tif]

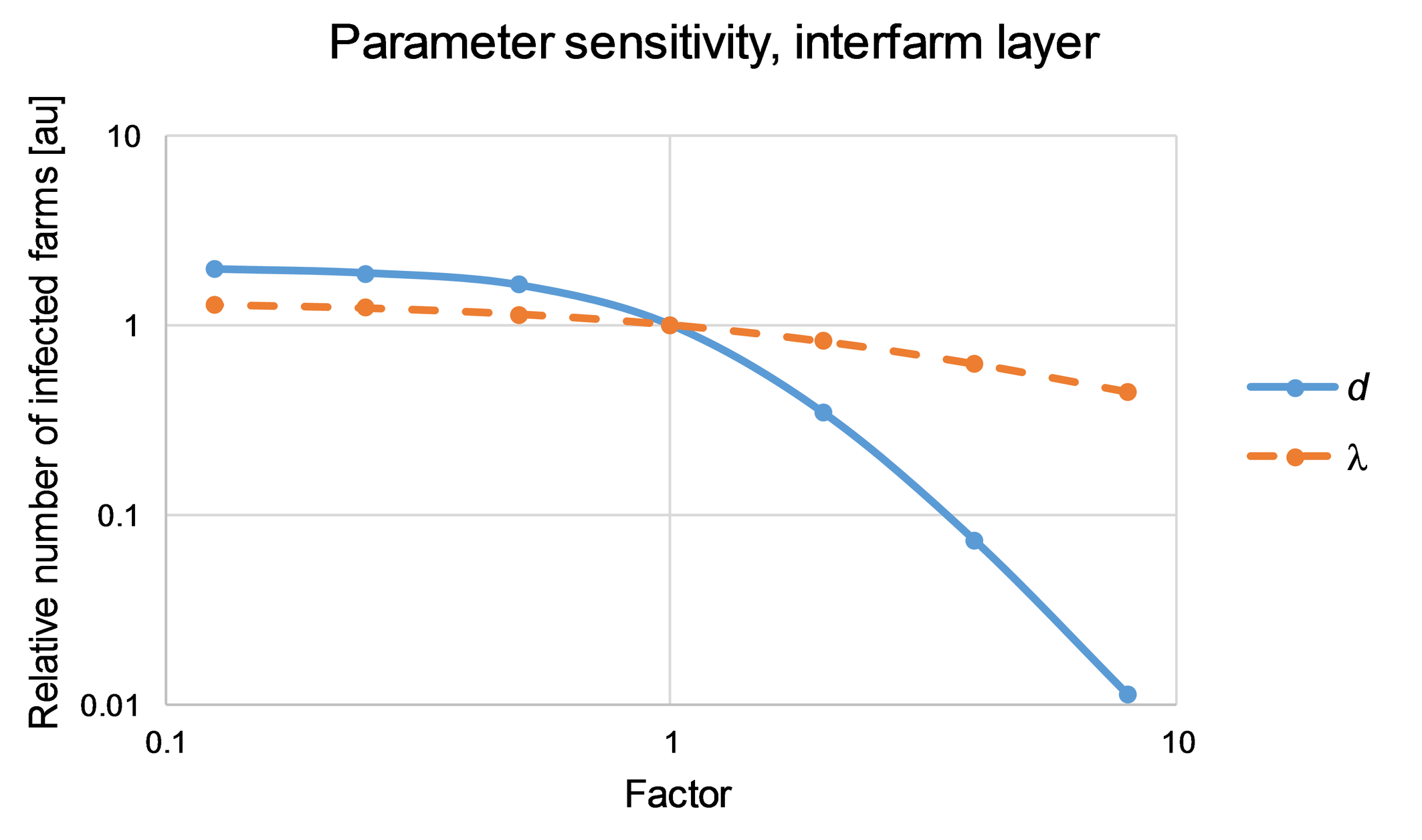

Supplement: S5 Fig — (TIF) [file pone.0232489.s007.tif]
